# Heterologous prime-boost immunization induces protection against dengue virus infection in cynomolgus macaques

**DOI:** 10.1128/jvi.00963-23

**Published:** 2023-10-17

**Authors:** Poonsook Keelapang, Chutitorn Ketloy, Chunya Puttikhunt, Rungtawan Sriburi, Eakachai Prompetchara, Malinee Sae-Lim, Bunpote Siridechadilok, Thaneeya Duangchinda, Sansanee Noisakran, Nicha Charoensri, Prapat Suriyaphol, Piyanan Suparattanagool, Utaiwan Utaipat, Promsin Masrinoul, Panisadee Avirutnan, Juthathip Mongkolsapaya, Gavin Screaton, Prasert Auewarakul, Suchinda Malaivijitnond, Sutee Yoksan, Prida Malasit, Kiat Ruxrungtham, Rojjanaporn Pulmanausahakul, Nopporn Sittisombut

**Affiliations:** 1 Department of Microbiology, Faculty of Medicine, Chiang Mai University, Chiang Mai, Thailand; 2 Center of Excellence in Vaccine Research and Development, Faculty of Medicine, Chulalongkorn University, Bangkok, Thailand; 3 Department of Laboratory Medicine, Faculty of Medicine, Chulalongkorn University, Bangkok, Thailand; 4 Molecular Biology of Dengue and Flaviviruses Research Team, Medical Molecular Biotechnology Research Group, National Center for Genetic Engineering and Biotechnology, National Science and Technology Development Agency, Pathumthani, Thailand; 5 Division of Dengue Hemorrhagic Fever Research, Siriraj Center of Research Excellence in Dengue and Emerging Pathogens, Faculty of Medicine Siriraj Hospital, Mahidol University, Bangkok, Thailand; 6 Frontier Biodesign and Bioengineering Research Team, National Center for Genetic Engineering and Biotechnology, Pathumthani, Thailand; 7 Center for Research and Development of Medical Diagnostic Laboratories, Faculty of Associated Medical Sciences, Khon Kaen University, Khon Kaen, Thailand; 8 Siriraj Informatics and Data Innovation Center, Faculty of Medicine Siriraj Hospital, Mahidol University, Bangkok, Thailand; 9 Clinical Epidemiology Unit, Faculty of Medicine, Khon Kaen University, Khon Kaen, Thailand; 10 Research Institute for Health Sciences, Chiang Mai University, Chiang Mai, Thailand; 11 Center for Vaccine Development, Institute of Molecular Biosciences, Mahidol University at Salaya, Nakhon Pathom, Thailand; 12 Wellcome Centre for Human Genetics, Nuffield Department of Medicine, University of Oxford, Oxford, United Kingdom; 13 Department of Microbiology, Faculty of Medicine Siriraj Hospital, Mahidol University, Bangkok, Thailand; 14 National Primate Research Center of Thailand, Chulalongkorn University, Bangkok, Thailand; 15 Princess Srisavangavadhana College of Medicine, Chulabhorn Royal Academy, Bangkok, Thailand; Lerner Research Institute, Cleveland, Ohio, USA

**Keywords:** dengue, prime-boost immunization, attenuated virus, virus-like particle, DNA vaccine

## Abstract

Currently licensed dengue vaccines do not induce long-term protection in children without prior dengue virus exposure. A better understanding of the mechanism by which the immune system prevents dengue virus infection is urgently needed to improve vaccine efficacy. In this study, the induction of protective antibody responses against dengue virus infection was tested in a non-human primate model using the heterologous prime-boost vaccination approach. Groups of cynomolgus macaques were immunized with a priming dose of attenuated dengue viruses and followed by two booster doses of virus-like particles in four monovalent arms, or in the tetravalent arm (prM + E)-expressing plasmids. At 1 month post-immunization, all macaques had elevated levels of neutralizing antibodies, and live viral challenges revealed an overall protective efficacy of 91% (40/44 macaques protected) against infection with clinical isolates. Breakthrough infections occurred in macaques with distinctive antibody profiles at the time of challenge: two macaques had the lowest neutralizing antibodies against the respective DENV-1 and -4 challenge strains among the respective groups, whereas two other DENV-4-infected macaques exhibited high levels of neutralizing and virus-binding antibodies. The ratio of antibodies recognizing a DENV-4-specific epitope and those that bound viral particles was at the lowest levels in the latter DENV-4-infected macaques, indicating an underrepresentation of antibodies targeting the serotype-specific epitope. Protection among macaques challenged with DENV-2 or -3 coincided with vigorous EDIII-binding antibody responses induced by booster immunization. A combination of attenuated viruses for priming and non-infectious particle-based antigens for boosting may be a more effective means of preventing dengue.

**IMPORTANCE:**

Currently licensed dengue vaccines do not induce long-term protection in children without previous exposure to dengue viruses in nature. These vaccines are based on selected attenuated strains of the four dengue serotypes and employed in combination for two or three consecutive doses. In our search for a better dengue vaccine candidate, live attenuated strains were followed by non-infectious virus-like particles or the plasmids that generate these particles upon injection into the body. This heterologous prime-boost immunization induced elevated levels of virus-specific antibodies and helped to prevent dengue virus infection in a high proportion of vaccinated macaques. In macaques that remained susceptible to dengue virus, distinct mechanisms were found to account for the immunization failures, providing a better understanding of vaccine actions. Additional studies in humans in the future may help to establish whether this combination approach represents a more effective means of preventing dengue by vaccination.

## INTRODUCTION

Dengue viruses (DENV) are a group of enveloped, mosquito-transmitted, positive-stranded RNA viruses that belong to the family *Flaviviridae*. There are four serotypes of dengue viruses (DENV-1-4), which differ from one another by about 25%–40% in the envelope protein sequences (1). Dengue viruses commonly cause asymptomatic infections in humans and monkeys. In a minority of infected individuals, a spectrum of illnesses, including undifferentiated fever, dengue fever, dengue hemorrhagic fever, and unusual manifestations (2), can be fatal and continue to pose a great economic burden (3). Two licensed tetravalent live-attenuated vaccines (LAVs), Dengvaxia (CYD-TDV) and TAK-003 (DENVax or Qdenga), are highly effective in reducing dengue severity and the need for hospitalization (4 – 6). However, neither vaccine induces protective immune responses in young children without prior natural exposure to the dengue virus (6 – 9). Dengvaxia is known to be minimally effective in preventing asymptomatic dengue virus infection (10), has no efficacy against DENV-2 in <9-year-old children, and is effective only against DENV-4 in dengue virus-seronegative recipients. TAK-003 is highly effective against DENV-2 but less efficacious against other serotypes (6, 11, 12).

Currently, Dengvaxia is recommended for use in dengue-seropositive children and adults (13). Recent data suggest that the minimum age of recipients can be lowered to 6 years, and the vaccination scheme can be shortened from three-dose to two-dose schedules without loss of efficacy (14). Selective vaccination of DENV-exposed persons, however, could potentially reduce the annual incidence of dengue by only 20%–30% as compared with 90% expected from Wolbachia-infected mosquito intervention (15). Together with the limited period of protection provided by Dengvaxia and TAK-003 (12, 16), these shortcomings necessitate further improvement of the LAV approach. A third tetravalent LAV candidate, TV003, currently being tested in a phase III clinical trial, is endowed with a different set of attenuating mutations as well as the non-structural protein coding sequences derived from three dengue serotypes. It induces high levels of neutralizing antibodies and CD4^+^ T cell responses against multiple dengue serotypes (17, 18) but causes a high incidence of transitory skin rashes (19), which may limit its use.

As all four dengue serotypes can cause severe diseases, one of the major goals in the development of tetravalent dengue LAV is to stimulate balanced immunity against all four serotypes (17, 20, 21). This goal is not always achievable, particularly among young and seronegative vaccine recipients, as shown by weak antibody responses in the plaque reduction neutralization test (PRNT) (6, 8, 11). Dengvaxia induces predominantly DENV-4 serotype-specific (homotypic) neutralizing antibodies in dengue virus-naive recipients, which explains the high efficacy of this vaccine against DENV-4 (11, 22). Similarly, the extraordinary efficacy of TAK-003 against DENV-2 is associated with high levels of neutralizing antibodies targeting DENV-2-specific epitopes and low levels of DENV-1, -3, and -4 neutralizing antibodies that mainly recognize cross-reactive epitopes (23). Another study showed that only 48% and 76% of single-dose TV003 recipients had tetravalent and trivalent homotypic-neutralizing antibody responses, respectively, and as high as 38% of all recipients were unable to mount a monovalent homotypic-neutralizing antibody response to DENV-1 (17). These findings suggest that the tetravalent LAV approach, even when employed in two-dose (TAK-003) or three-dose (Dengvaxia) schedules, is unlikely to stimulate a balanced homotypic-neutralizing antibody response against all dengue serotypes.

The inability of LAV-based immunization to induce balanced homotypic-neutralizing antibody responses in seronegative children is reflected in the test results from live viral challenge studies. The rise in neutralizing antibodies after live viral challenge signifies that vaccination failed to mount solid protection (sterilizing immunity) (24); hence, monitoring of neutralizing antibody levels can be used to assess vaccine efficacy. Using a fourfold or greater increase in neutralizing antibody titer after live viral challenge as the criterion for breakthrough infection, a single dose of Dengvaxia was found to prevent dengue virus infection in only 1 out of 23 rhesus macaques (*Macaca mulatta*) that seroconverted against the challenge serotypes (25). At 1 month after the second dose, TAK-003 (in three different formulations) was shown to induce protection against the wild-type DENV-2 infection in four out of six cynomolgus macaques (*Macaca fascicularis*) but failed to protect five out of six macaques from wild-type DENV-1, -3, or -4 viruses (26). In a human challenge study employing a partially attenuated DENV-2 strain, rDEN2Δ30, up to 43% of flavivirus-naive TV003 recipients showed a fourfold or more increase in the neutralizing antibody titer (27). In contrast, these vaccines are highly effective in preventing post-challenge viremia (25 – 27), which suggests that neutralizing antibody rise may be a more accurate metric for predicting the outcome of clinical trials based on challenge study results.

An inherent difficulty in using multiple doses of dengue LAV is related to its sensitivity to antibody-mediated neutralization, which necessitates long intervals between repeated doses to achieve the boosting effect (21, 28). Substitution of LAV with non-infectious viral particles for boosting may help shorten the priming-to-boosting interval, with the added benefit of tailoring particles with the desired properties. The main goal of this study was to assess whether a heterologous LAV-primed/non-infectious particle-boosted approach could induce protective antibody responses against dengue virus infection in cynomolgus macaques. We designed a 4-month three-injection immunization scheme employing non-infectious boosting particles. A greater than or equal to fourfold increase in post-challenge neutralizing antibodies was used as the criterion for breakthrough infection. In separate monovalent and tetravalent approaches, LAV-primed macaques were boosted twice at 2 and 3 months after the priming injection by employing particle-based immunogens in the form of virus-like particles (VLPs) or envelope protein-expressing plasmids. Moreover, the DENV-1 and -2 LAV components had been genetically modified by the introduction of a prM cleavage-enhancing mutation to increase the proportion of mature viral particles (29 – 31), which may more closely resemble the majority of circulating virions during dengue virus infection in humans (32). Antibody responses to immunization were determined by PRNT together with binding and blockade-of-binding assays to evaluate antibody properties, including the recognition of the whole viral particle, the receptor-binding domain of the major envelope protein E, and the E-associated serotype-specific epitopes (33).

## MATERIALS AND METHODS

### Dengue viruses

Prototypic dengue viruses (DENV-1 strain 16007, DENV-2 strain 16681, DENV-3 strain 16562, and DENV-4 strains 1036 and C0036/06) were provided by Drs. Bruce Innis and the late Ananda Nisalak, Armed Forces Research Institute of Medical Sciences, Bangkok for use in PRNT. Clinical dengue strains (DENV-1 strain 03-0398, DENV-2 strain 03-0420, DENV-3 strain 06/129, and DENV-4 strain 2-0201-5) were isolated from pediatric patients in Thailand during 2003−2006 (31). Viruses were propagated in Vero cells using Minimum Essential Medium (MEM, Invitrogen) supplemented with 2% fetal bovine serum (FBS) and glutamine-penicillin-streptomycin solution at 37°C in humidified air regulated to 5% CO_2_, and stored in 20% (vol/vol) FBS at −70°C. Infectious viruses were quantified using a focus immunoassay titration method (34). Dengue viruses were purified from the culture media by precipitating with 8% (wt/wt) polyethylene glycol 8000 (Sigma) and 150 mM NaCl, collected by centrifugation at 9,500 rpm, 4°C for 50 min using an F0685 fixed angle rotor and Allegra 64R centrifuge (Beckman Coulter) and then subjected to successive centrifugations employing a 22% sucrose cushion and a 10%–35% potassium tartrate-glycerol gradient [both at 32,000 rpm, 4°C, 2 h using the SW41 rotor and an ultracentrifuge (Optima L-100 XP, Beckman Coulter)] as described previously (30). Purified viruses were stored in phosphate-buffered saline (PBS) in the presence of 20% (vol/vol) glycerol at −20°C.

### Live-attenuated dengue viruses

Live-attenuated chimeric dengue viruses were generated from 16681-3pm, a recombinant strain harboring the three attenuating point mutations of strain 16681 PDK-53 (35), by using a previously described (prM + E)-coding region replacement scheme (31). The (prM + E)-coding region of strains cD1-4pm, cD3-3pm, and cD4-3pm were derived from DENV-1 strain 03-0398, DENV-3 strain 06/129, and DENV-4 strain 2-0201-5, respectively. Strain cD2-4pm contained a DENV-2 (prM + E) consensus sequence (36). Virally encoded codons in the (prM + E)-coding region of cD2-4pm and cD3-3pm were substituted with human-optimized codons. Codon optimization leads to increased plasmid stability during propagation in *Escherichia coli,* increased viral focus size, and enhanced virus replication (37). The pr-M cleavage-enhancing mutation, prE203A or its equivalent (29), was also introduced into strains cD1-4pm and cD2-4pm. In the 16681-3pm genetic background, the prD203A mutation has been shown to result in a prM cleavage efficiency of 87.5% for cD1-4pm, which was comparable to its known effect (90.9%) in strain 16681 (30, 31). The chimeric strain cD4-(2 + 1)pm containing only the NS1 Gly53Asp and NS3 Glu250Val attenuating point mutations together with the prE203A mutation was employed in the tetravalent arm instead of cD4-3pm. The recombinant virus MBU-1 was derived from a DENV-3 LAV strain, 16562 PGMK-30/PDK-4 (38), using the protocol as described previously (39).

### Virus-like particles

DENV-2 partially mature VLP (pVLP) was obtained as described previously (40). Mature virus-like particles (mVLP) were generated by transfecting a mosquito-derived cell line, C6/36, with (M + E)-expressing plasmids. The (M + E) coding sequences were derived from the following clinical strains: DENV-1 strain 03-0398, DENV-2 strain 03-0420, DENV-3 strain 06/129, and DENV-4 strain 1228, with modifications of selected histidine and non-histidine residues intended to minimize acid-induced conformational changes during export (Table S1). The (M + E) coding sequences were cloned into the insect cell expression vector pIE1-SP-prME (41). Transfected cells stably expressing mVLP at high yield were selected with blasticidin, and then expanded in Leibovitz’s L15 medium (Invitrogen) supplemented with 10% FBS and 0.26% tryptose phosphate broth (Sigma Chemical) at 29°C in ambient air. VLPs were concentrated from the culture media by ultrafiltration using a 500 kDa cut-off membrane (Biomax-500 PB, Amicon), partially purified by sucrose density gradient centrifugation [20%–55% sucrose in 120 mM NaCl, 20 mM Tris-HCl pH 8.0, 1 mM EDTA (NTE) at 32,000 rpm, 4°C for 2 h using a SW41 rotor], and purified further by rate zonal centrifugation (5%–25% sucrose in NTE buffer at 25,000 rpm, 4°C for 3 h using a SW41 rotor in an Optima L100 XP Beckman Coulter ultracentrifuge). VLPs were stored in PBS in the presence of 20% (vol/vol) glycerol at −20°C. Purified VLPs were visualized by negative staining using 1% uranyl acetate and a transmission electron microscope (Jeol, JEM-2200FS) (Fig. S1).

### DNA vaccine candidates

A set of (prM + E)-expressing plasmids was generated based on pCMVkan (42), employing the DENV-1, -2, -3, and -4 consensus sequences derived from 133, 124, 54, and 65 GenBank-deposited viral sequences, respectively, as described previously (36). Human-optimized codons were employed in the (prM + E)-coding region for all plasmids. Upon transfection of the flavivirus (prM + E)-expressing plasmids into mammalian cells, intracellular assembly of the expressed prM and E proteins into particulate virus-like forms and the secretion of these particles into the culture media have been observed in a number of studies (43 – 46).

### Macaques

Male and female cynomolgus macaques (*Macaca fascicularis*), 3–6 years of age, were screened using hematological and liver function tests and a plaque/focus reduction neutralizing antibody test with the 50% reduction endpoint (PRNT_50_ or FRNT_50_) for the absence of serum antibodies against dengue viruses and Japanese encephalitis virus. At enrollment, the median body weight of female macaques was 2.72 kg (range 2.36–5.68 kg, *n* = 22) as compared to 4.53 kg (range 2.55–7.84 kg, *n* = 41) of male macaques (*P* < 0.0001, Fig. S2A). Macaques were housed in individual cages equipped with insect screens and provided with standard water and food allowances at the Indonesian facility. At the National Primate Research Center of Thailand-Chulalongkorn University (Thailand site), macaques were housed in individual cages in the Animal Biosafety Level-2, negative air-pressure room at a temperature of 25°C ± 2°C and relative humidity 60% ± 10%. The monkeys were fed with standard monkey chow in the morning, fresh fruits and vegetables in the afternoon, and drinking water *ad libitum*. At both sites, body weight and temperature were measured every other day after immunization or live viral challenge. Blood samples were taken from the femoral vein under ketamine hydrochloride-induced anesthesia, processed, and stored at −80°C.

### Immunization and challenge

In the monovalent immunization arm, groups of macaques received a priming subcutaneous injection of 1 × 10^5^ focus-forming unit (FFU) of cD1-4pm (*n* = 6), cD2-4pm (*n* = 6), or MBU-1 (*n* = 3); 5 × 10^5^ FFU of cD3-3pm (*n* = 2); or 1 × 10^6^ FFU of cD4-3pm (*n* = 3). After 2 months, two booster doses of 10 µg VLP of the same serotype were injected subcutaneously in the presence of Addavax adjuvant (InvivoGen) at 1-month intervals. In the tetravalent immunization arm, a combination of live-attenuated chimeric viruses—1 × 10^5^ FFU of cD1-4pm and cD2-4pm, 3 × 10^5^ FFU of cD3-3pm, and 6 × 10^5^ FFU of cD4-(2 + 1)pm (tLAV)—was injected subcutaneously at one site to 24 macaques (12 of each sex), followed by two 500 µg/serotype booster doses of the (prM + E)-expressing plasmid mixture administered by intradermal injection/*in vivo* electroporation (Ichor Medical Systems, CA, USA) at 1-month intervals (36). Following the priming injection, blood samples were taken on alternate days during the subsequent 14-day period for the detection of viremia. Viremia was negligible after LAV and tLAV injections (Fig. S3A through S3H).

At 1 month after the second booster injection (day 120), groups of macaques in each immunization arm were challenged by subcutaneous injection of 1 × 10^5^ PFU of clinical dengue isolates for each of the four dengue serotypes (DENV-1 strain 03-0398, DENV-2 strain 03-0420, DENV-3 strain 06/129, or DENV-4 strain 2-0201-5). Additional groups of three macaques (one female and two males) in the control arm were injected with clinical dengue isolates for the validation of challenge viruses. Blood samples were taken on alternate days during the subsequent 14-day period for the detection of viremia and viral RNAemia, and at 2 and/or 4 weeks for measuring the neutralizing antibody. A fourfold or more increase in the PRNT_50_ titer against the injected serotype from levels observed 2 days before the challenge (day 118) was used to indicate breakthrough infection. Blood samples were also obtained for hematological investigations on days 8, 30, 74, 104, 128, and 150, and for liver function tests on days 30 and 150. Except for the DENV-4 monovalent prime-boost and challenge study, all other studies were performed concurrently in Bogor, Indonesia. All monkeys in this study did not display any sign of illnesses related to dengue virus infection after receiving the LAV or challenge viruses. Bleeding was not performed on consecutive days to minimize the stress-induced anorexia and stereotypic behaviors. When occurred, these disorders were relieved after rest and provision of vitamin supplements and topical antibiotics.

### Detection of viremia and viral RNAemia

Infectious viruses in monkey plasma were quantified by direct focus formation on Vero cell monolayers. Plasma samples were diluted 1:5 with MEM supplemented with 2% FBS, and 50 µL was transferred onto cell monolayer in quadruplicate wells in a 96-well cell culture plate (Corning) and incubated with intermittent manual shaking at 37°C for 2 h. Diluted plasma and/or clot were then removed from the wells, and replaced with an overlayer of MEM containing 2% FBS, penicillin-streptomycin-glutamine solution, and 1.2% carboxymethylcellulose (Sigma Chemicals, St. Louis, MO, USA). After 3 days of incubation at 37°C in humidified air regulated to 5% CO_2_, cell monolayers were fixed with 3.7% formaldehyde in PBS and permeabilized with 2% Triton X-100 in PBS. Monoclonal antibody 4G2 specific for a flavivirus group epitope was used in the immunological staining of virus-infected foci as in the focus immunoassay titration method. The limits of detection were determined by reconstitution of strain 16681 in naive monkey plasma in three separate experiments to be 3, 8, and 8 FFU/mL for the range of input viruses of 1–14 FFU/mL.

RNA was extracted from plasma samples using a MagNA Pure Compact nucleic acid isolation kit (Roche) according to the manufacturer’s protocol. Viral RNAemia was detected by the Taqman-based quantitative RT-PCR method (47). Sequence modifications were introduced into the primers and probes to match clinical dengue strains employed in the challenge study; the sequences of primers and probes are available upon request. Genomic RNA extracted from a concentrated preparation of DENV-1 strain 03-0398 and purified *in vitro* transcripts of viral cDNA clones encoding the 3′ untranslated region of DENV-2 strain 16681 (680 bases in length), DENV-3 strain 06-129 (2,046 bases), and DENV-4 strain 2-0201-5 (2,067 bases) were employed as the quantity controls (48). Undetectable level was depicted as 1 RNA copy/mL in graphical illustrations.

### Neutralization test

Dengue virus-neutralizing antibodies were quantified using the PRNT_50_ method according to a WHO protocol (49). The FRNT_50_ was performed essentially in the same manner as the PRNT_50_ (34). When the neutralizing activity was not detected at the first serum dilution of 1:10, an arbitrary titer of 5 was used in statistical analyses and displayed in graphs.

### Enzyme-linked immunosorbent assay

Enzyme-linked immunosorbent assay (ELISA) for the measurement of antibodies specific for dengue virus particles, NS1, recombinant S2 cell-derived E80, and *E. coli*-derived EDIII domains was performed concurrently for the four dengue serotypes essentially as described previously (40, 50). Briefly, serum or plasma samples were twofold serially diluted starting from 1:40. Bound antibodies were detected by using horse radish peroxidase (HRP)-conjugated rabbit anti-monkey IgG antibody (Sigma) and H_2_O_2_/tetramethylbenzidine substrate. Antigen-specific absorbance was derived by subtracting the absorbance of the antigen-coated/bovine serum albumin (BSA)-blocked well with the absorbance of the BSA-only well that was measured separately for each dilution of the sample. The endpoint titer (EPT) was determined as the reciprocal of the dilution that resulted in the antigen-specific absorbance of 0.300, or the highest values of naive macaque samples. A sample with less than the endpoint value at the first dilution (1:40) was given an arbitrary titer of 20. An anti-DENV EDIII monoclonal antibody, clone 2H12 (51), or pooled human convalescent sera was employed as a control for the comparison between plates. Pooled convalescent plasma and pooled seronegative donor plasma were used in all plates as the positive and negative controls, respectively.

### Blockade-of-binding ELISA

Neutralizing monoclonal antibodies specific for each dengue serotype—DENV-1, clone 1F4 (52); DENV-2, clone 3H5 (53); DENV-3, clone 8A1 (54, 55), and DENV-4, clone 5H2 (56)—and all serotypes—clones 513 and EDE-1 C10 (57, 58)—were purified from the cell culture media using protein A/G affinity chromatography. Clones 1F4, 5H2, 513, and EDE-1 C10 were generated based on published VH and VL sequences by cloning synthesized sequences in the human IgG expression vector pVitro1-dV-IgG1/λ and subsequently transfecting the recombinant plasmids into Expi293 cells (Thermo Fisher Scientific) as described previously (59). Purified antibodies were conjugated with HRP employing a conjugation kit (KPL SureLink HRP conjugation kit, SeraCare) according to the manufacturer’s protocol. Prior to use, HRP-conjugated antibodies were titrated by binding the serially diluted preparations to graded amounts of microtiter plate-bound, gradient-purified dengue virus particles; the amounts of antibody and virus employed in the blocking assay were adjusted empirically to achieve an absorbance reading of about one unit in the blocking assay.

The blockade-of-binding assay was performed as described previously (33). Briefly, a pre-titrated amount of purified virus particles in PBS, pH 7.4, was applied to the wells of a 96-well microtiter plate (Maxisorp, Nunc) overnight at 4°C. Unbound viruses were removed, and the non-specific binding sites were blocked with 1% BSA (heat shock fraction, pH 7, Sigma-Aldrich) in PBS at room temperature for 1 h. Serum or plasma samples were serially diluted with 0.5% BSA in PBS and 50 µL was applied onto the virus-coated/BSA-treated wells in parallel with graded concentrations (0.2 ng/mL–50 µg/mL) of an unconjugated antibody at room temperature (or 37°C in the case of 8A1) for 1.5 h. As controls, BSA-treated wells (background control) and virus-coated/BSA-treated wells (binding control) were present in triplicate in all plates. After removing unbound sera/plasma by washing with 0.05% Tween 20 in PBS, the corresponding HRP-conjugated antibody in 0.5% BSA in PBS was added to all wells at room temperature for 1 h. Following additional washings with 0.05% Tween 20 in PBS, a substrate mixture containing H_2_O_2_ and 3,3′,5,5′-tetramethyl benzidine (Novex TMB single solution, Life Technologies) was added, and the absorbance was measured at 450 nm. The percent reduction in the absorbance of the experimental well as compared to the binding control well was calculated. A standard curve was then generated from the percent reduction of graded concentrations of unconjugated antibody. The relative concentration of the epitope-blocking activity of a sample was determined by interpolation of the standard curve using the percent reduction at the first dilution (1:40).

### Statistical analysis

Body weight and PRNT_50_ titer were compared between male and female macaques using the two-tailed Mann-Whitney test with computation for the exact *P*-value. For detecting the reduction of post-challenge viral RNAemic days as a result of immunization, the viral RNAemic days were compared employing the one-tailed Mann-Whitney test. *P* ≤ 0.05 was considered significant. Analyses were performed using GraphPad Prism, version 9.5.1.

## RESULTS

### Heterologous prime-boost immunization-induced protection against live dengue virus challenge in cynomolgus macaques

**Fig 1:**
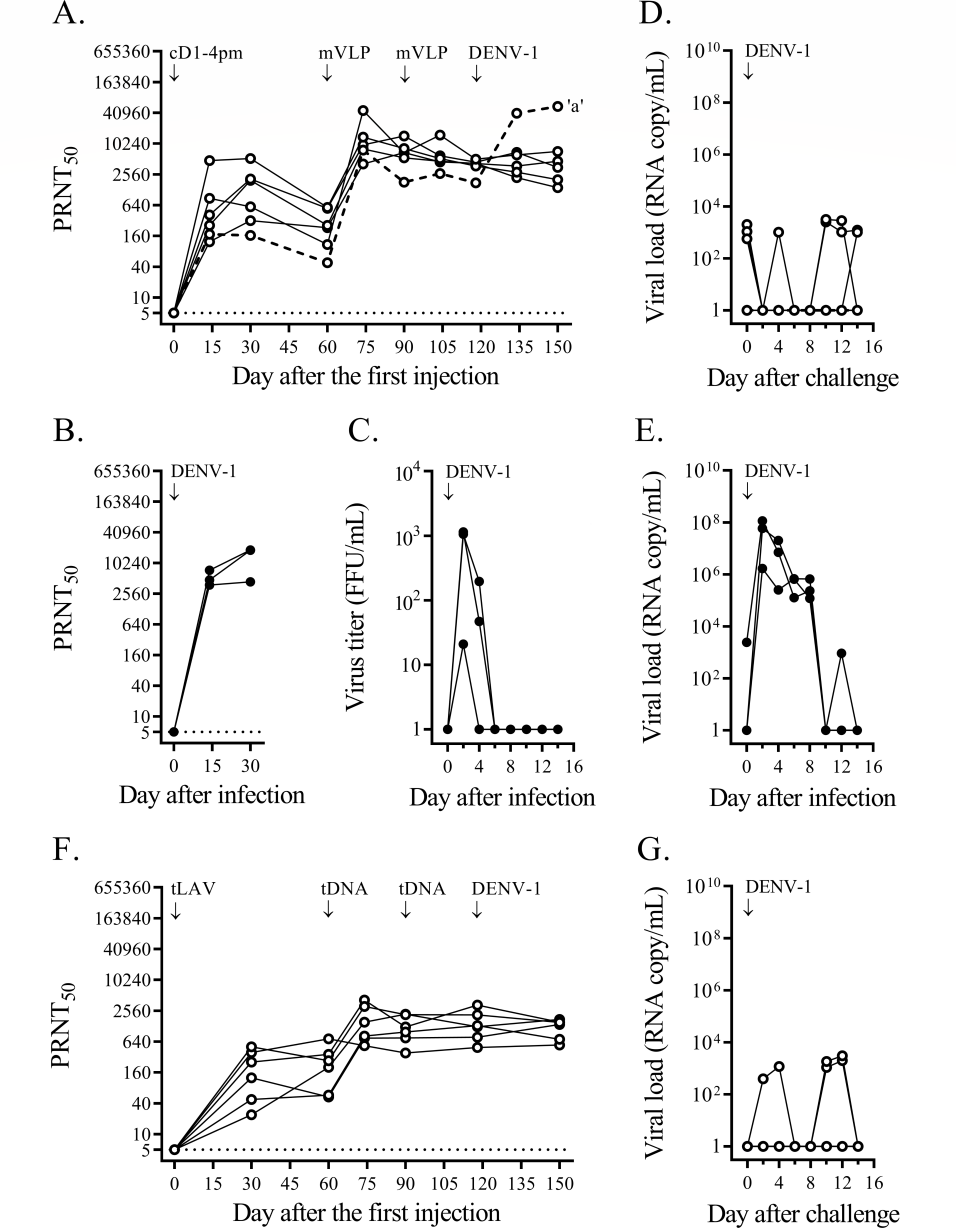
Heterologous prime-boost immunizations against DENV-1 challenge. (**A**) A group of six cynomolgus macaques was immunized with cD1-4pm and two doses of DENV-1 mVLP, as indicated by the arrows. One month after the second mVLP injection, macaques were challenged subcutaneously with 1 × 10^5^ FFU of the DENV-1 clinical strain 03-0398. PRNT_50_ titers against the DENV-1 prototype are shown at different time points for each macaque. The limit of detection for the neutralizing antibody activity at the first dilution of the blood sample (1:10) was 5, as indicated by the dotted line. A macaque with a more than fourfold increase in the neutralizing antibody titer after challenge (macaque ‘a’) is indicated by a dashed line. (**B**) PRNT_50_ titer against DENV-1 for three non-immunized control cynomolgus macaques infected with the same dose of strain 03-0398 as in panel (**A**). (**C**) DENV viremia assessed in plasma samples of DENV-1-infected, immunized macaques (panel B) by direct focus formation in the Vero cell monolayer. (**D and E**) DENV-1 RNAemia determined by quantitative RT-PCR from blood samples taken on alternate days after the challenge of mVLP-immunized macaques (panel A) and after infection of non-immunized controls (panel B), respectively. (**F**) PRNT_50_ titer for a group of six cynomolgus macaques immunized with a mixture of four chimeric live attenuated virus strains (tLAV) and two doses of tetravalent (prM + E)-expressing plasmids (tDNA), and then challenged with strain 03-0398 as in the monovalent approach. (**G**) DENV-1 RNAemia determined by quantitative RT-PCR from blood samples taken on alternate days after the challenge from tLAV-immunized macaques (panel F).

**Fig 2:**
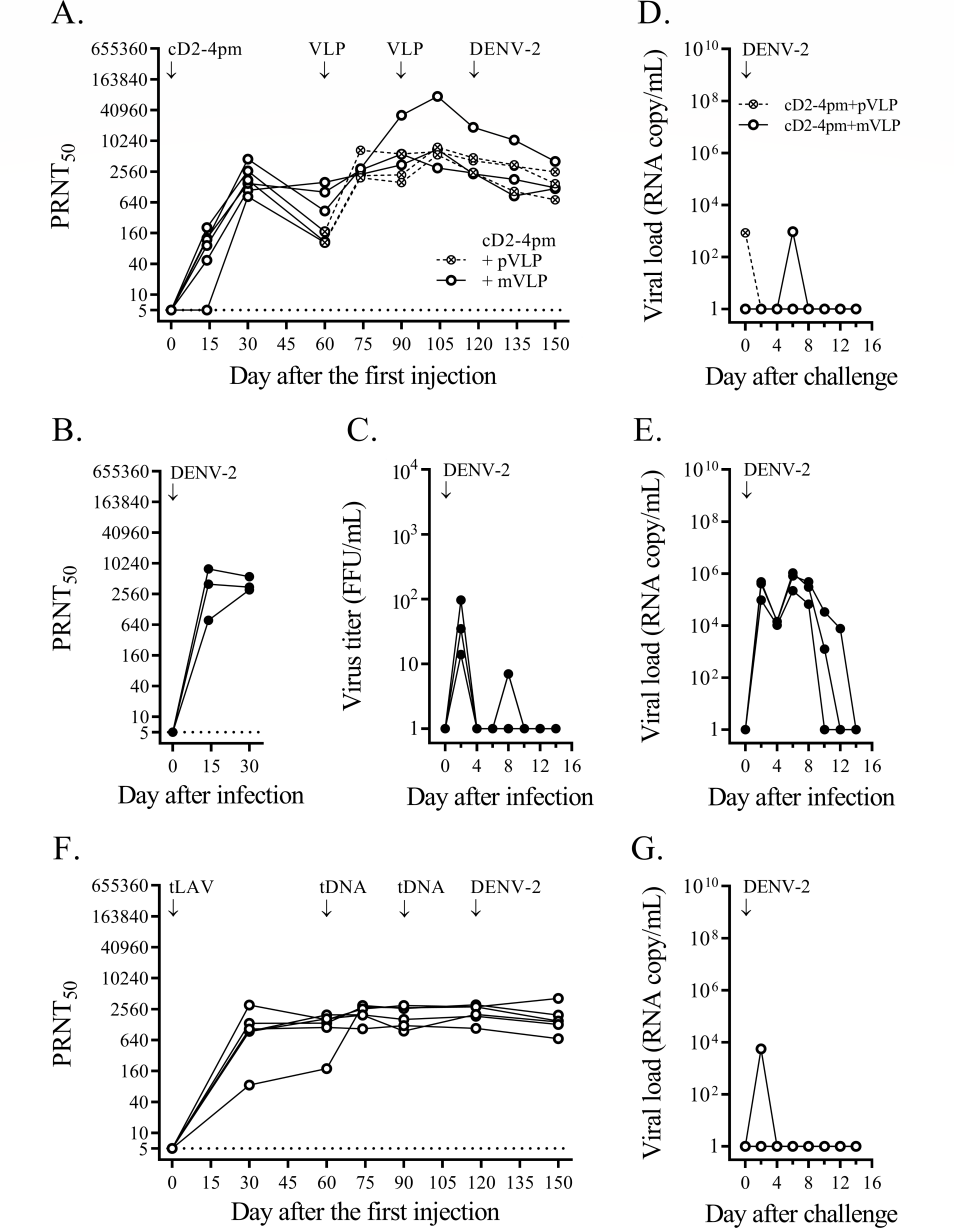
Heterologous prime-boost immunizations against DENV-2 challenge. (**A**) A group of six cynomolgus macaques was immunized with cD2-4pm and then separated into two subgroups to receive two doses of DENV-2 mVLP (empty circles and solid lines) or partially mature VLP (crossed circles and dashed lines). One month after the second VLP injection, they were challenged with 1 × 10^5^ FFU of the DENV-2 clinical strain 03-0420. PRNT_50_ titers against the DENV-2 prototype are shown for each macaque. The limit of detection for the neutralizing antibody activity at the first dilution of the blood sample (1:10) was 5, as indicated by the dotted line. (**B**) PRNT_50_ titer against DENV-2 for three non-immunized control cynomolgus macaques infected with the same dose of strain 03-0420 as in panel (**A**). (**C**) DENV viremia assessed from plasma samples of DENV-2-infected non-immunized control macaques (panel B) by direct focus formation in the Vero cell monolayer. (**D and E**) DENV-2 RNAemia determined by quantitative RT-PCR from blood samples taken on alternate days after the challenge of immunized macaques (panel A) and in non-immunized controls after infection (panel B), respectively. (**F**) PRNT_50_ titer against DENV-2 for six cynomolgus macaques immunized with a mixture of four chimeric LAV strains (tLAV) and two doses of tetravalent (prM + E)-expressing plasmids (tDNA), and then challenged with strain 03-0420 as in the monovalent approach. (**G**) DENV-2 RNAemia determined by quantitative RT-PCR from blood samples taken on alternate days after the challenge from tLAV-immunized macaques (panel F).

**Fig 3:**
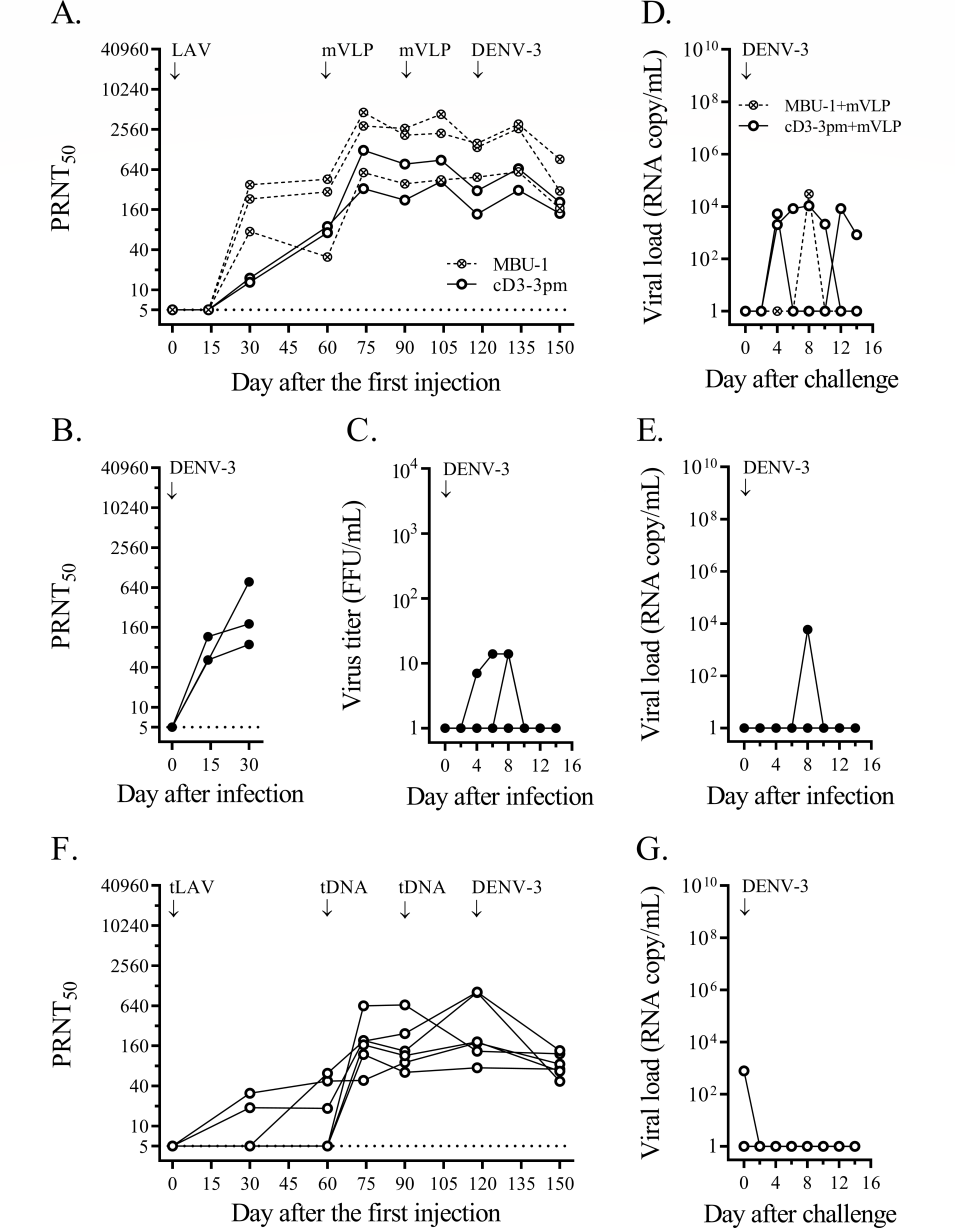
Heterologous prime-boost immunizations against DENV-3 challenge. (**A**) Two groups of three and two cynomolgus macaques were immunized with MBU-1 (crossed circles and dashed lines) and cD3-3pm (empty circles and solid lines), respectively, which then received two doses of DENV-3 mVLP. One month after the second VLP injection, they were challenged with 1 × 10^5^ FFU of DENV-3 clinical strain 06-129. PRNT_50_ titers against the DENV-3 prototype are shown for each macaque. The limit of detection for neutralizing antibody activity at the first dilution of the blood sample (1:10) was 5, as indicated by the dotted line. (**B**) Three non-immunized control cynomolgus macaques were infected with the same dose of strain 06-129 as in panel (**A**) and the PRNT_50_ titers against DENV-3 were determined. (**C**) DENV viremia was assessed in plasma samples of non-immunized control macaques (panel B) by direct focus formation in the Vero cell monolayer. (**D and E**) DENV-3 RNAemia was determined by quantitative RT-PCR from blood samples taken on alternate days after the challenge of immunized macaques (panel A) and after infection of non-immunized controls (panel B), respectively. (**F**) Six cynomolgus macaques were immunized with a mixture of four chimeric LAV strains (tLAV) and two doses of tetravalent (prM + E)-expressing plasmids (tDNA), and then challenged with strain 06-129 as in the monovalent approach. PRNT_50_ titers against the DENV-3 prototype are shown for each macaque. (**G**) DENV-3 RNAemia was determined by quantitative RT-PCR in blood samples taken on alternate days after the challenge from tLAV-vaccinated macaques (panel F).

The heterologous prime-boost immunization involved four monovalent arms and a tetravalent arm. In the monovalent approach, groups of cynomolgus macaques received, for each dengue serotype, a priming injection of live attenuated virus plus two boosting doses of VLP, and, after a month, were challenged with a clinical dengue isolate (Fig. 1A, 2A, 3A, and 4A). Immunization failure to protect against virus infection was recorded as a fourfold or greater increase in the neutralizing antibody titer against the challenge serotype within 4 weeks after the challenge.

**Fig 4:**
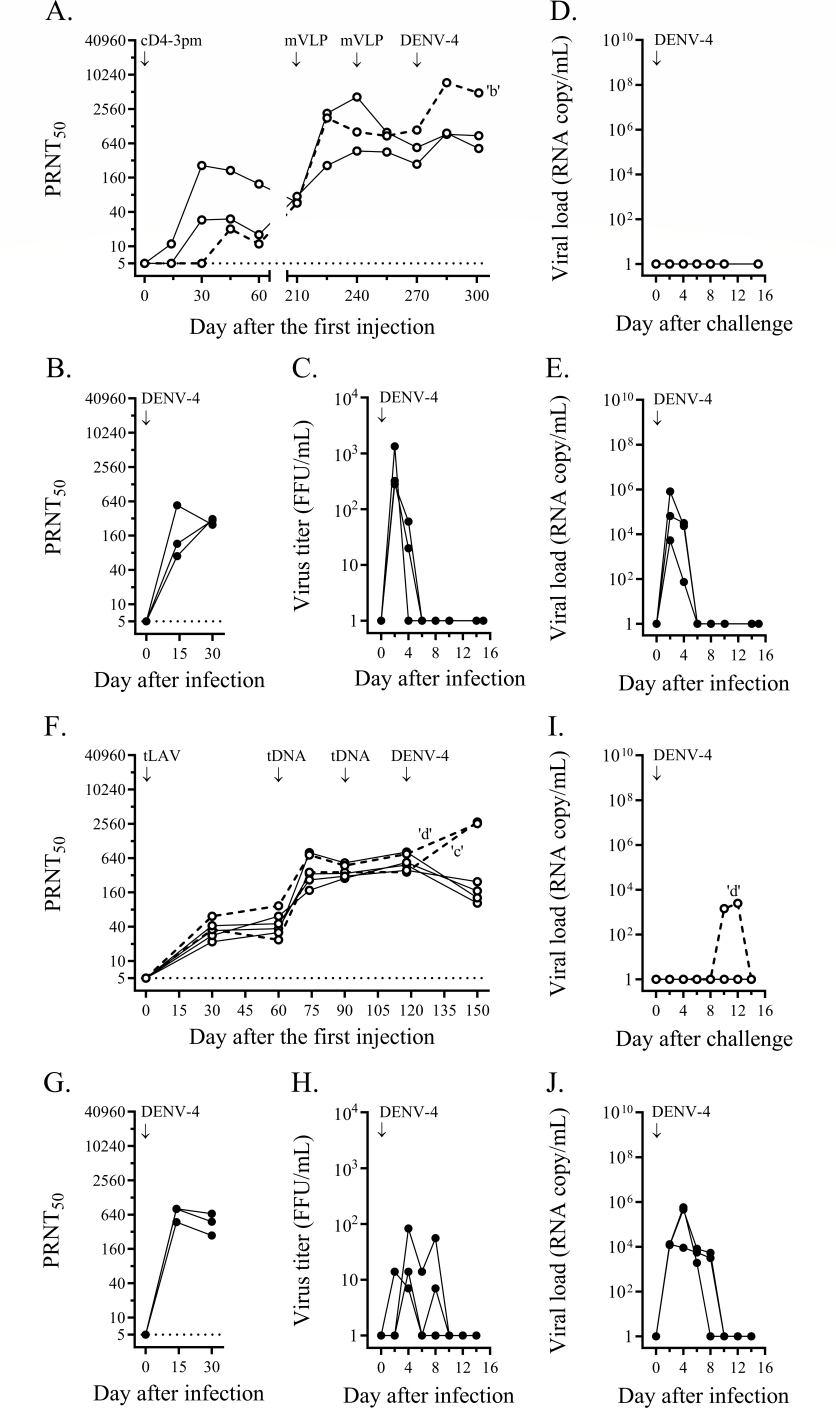
Heterologous prime-boost immunizations against DENV-4 challenge. (**A**) Three cynomolgus macaques were immunized with cD4-3pm, and after a 7-month interval, two doses of DENV-4 mVLP were administered. After 1 month, they were challenged with 1 × 10^5^ FFU of DENV-4 clinical strain 2-0205-1. PRNT_50_ titers against the DENV-4 prototype are shown for each macaque. The limit of detection for neutralizing antibody activity at the first dilution of the blood sample (1:10) was 5, as indicated by the dotted line. A macaque with a greater than fourfold increase in neutralizing antibody titer after the challenge (macaque ‘b’) is indicated by a dashed line. (**B**) Three non-immunized control cynomolgus macaques at the facility in Thailand were infected with the same dose of strain 2-0205-1 as in panel (**A**) and the PRNT_50_ titers against DENV-3 were determined. (**C**) DENV viremia was assessed in plasma samples of non-immunized control macaques (panel B) by direct focus formation in the Vero cell monolayer. (**D and E**) DENV-4 RNAemia was determined by quantitative RT-PCR from blood samples taken on alternate days after the challenge of immunized macaques (panel A) and after infection of non-immunized controls (panel B), respectively. (**F**) Six cynomolgus macaques were immunized with a mixture of four chimeric LAV strains (tLAV) and two doses of tetravalent (prM + E)-expressing plasmids (tDNA), and then challenged with strain 2-0205-1 as in the monovalent approach. Immunized macaques with a fourfold or greater increase in neutralizing antibody titer after the challenge (macaques ‘c’ and ‘d’) are indicated by dashed lines. (**G**) Three non-immunized control cynomolgus macaques at the facility in Indonesia were infected with the same dose of strain 2-0205-1 as in panel (**A**) and the PRNT_50_ titers against DENV-4 prototype were determined. (**H**) DENV viremia was assessed in plasma samples of macaques (panel G) by direct focus formation in the Vero cell monolayer. (**I and J**) DENV-4 RNAemia was determined by quantitative RT-PCR from blood samples taken on alternate days after challenge of immunized macaques (panel F) and after infection of non-immunized controls (panel G). A monkey, ‘d’, with RNAemia is indicated.

In the first monovalent immunization experiment, immunization with cD1-4pm and two subsequent doses of DENV-1 mVLP induced vigorous neutralizing antibody responses against DENV-1 (Fig. 1A). In contrast, the control group showed a rapid increase in neutralizing antibodies after DENV-1 injection, reaching a plateau within a month (Fig. 1B) and a brief period of viremia (Fig. 1C). One month after the second booster dose, the mean neutralizing antibody titer against DENV-1 in immunized macaques was comparable (less than fourfold difference) to the plateau level in DENV-1-infected control macaques (Fig. 1A and C). Based on the stable levels of neutralizing antibody in the 4-week period after the challenge, five out of six immunized macaques were protected against DENV-1 infection, whereas infected macaque ‘a’ that had the lowest level of neutralizing antibody against DENV-1 at the time of challenge was not protected (Fig. 1A). While viremia was not detected in any of the six macaques during the 2-week post-challenge period (Fig. S3I), viral RNAemia was about three to five orders of magnitude lower in immunized macaques than in control macaques (Fig. 1D vs Fig. 1E). There was a total of 10 viral RNAemic days in six immunized macaques vs 15 days in the control group (mean duration of 1.7 vs 5.0 days, *P* = 0.036). The sporadic detection of low levels of viral particles following the live virus challenge indicated that prime-boost immunization suppressed virus multiplication and abrogated DENV-1 infection in the majority of immunized macaques.

In the monovalent immunization against DENV-2, six macaques were injected with cD2-4pm and then boosted with either pVLP, which comprised pleomorphic particles resembling club-shaped dengue virus particles (40, 60), or mVLP. Similar levels of neutralizing antibodies were observed in the two immunized subgroups 1 month after the second booster injection (Fig. 2A), which were also similar to the levels in the control macaques injected with wild-type virus (Fig. 2B). Low levels of infectious virus were detected in the control group (Fig. 2C). Challenge with a clinical DENV-2 strain resulted in decreased neutralizing antibody titers and the absence of viremia in both immunized subgroups (Fig. 2A; Fig. S3J), indicating that none of the immunized macaques was infected with the challenge virus. With a total of two viral RNAemic days in all immunized macaques compared to 15 days in the three control macaques (Fig. 2D and E), the duration of viral RNAemia following challenge was significantly reduced in these monovalently primed-boosted macaques compared to the control macaques (*P* = 0.012).

In the third monovalent immunization arm, two subgroups of three and two macaques were injected with MBU-1, an attenuated DENV-3 strain, and cD3-3pm, a (prM + E)-chimeric DENV-2 virus containing the attenuating mutations of 16681 PDK-53, respectively (Fig. 3A). Delayed neutralizing antibody responses to LAV priming occurred in both subgroups (Fig. 3A), in contrast to the rapid response of naive macaques to infection with the wild-type DENV-3 clinical strain (Fig. 3B). A booster injection of mVLP increased the levels of neutralizing antibodies in all five macaques (Fig. 3A). As observed in the previous monovalent immunization arms (Fig. 1A and 2A), the second booster dose did not enhance antibody levels (Fig. 3A). Following challenge with a clinical DENV-3 strain, none of these immunized macaques showed a fourfold or more increase in the neutralizing antibody titer or viremia (Fig. 3A; Fig. S3K). One of the MBU-1-primed/mVLP-boosted macaques (Fig. 3D) and one of the control macaques (Fig. 3E) showed viral RNAemia after the challenge. In contrast, there were seven viral RNAemic days in the two macaques that received cD3-3pm and mVLP boosts (Fig. 3D), indicating a prolonged clearance of viral particles in the latter subgroup.

The fourth monovalent immunization arm testing the protection against DENV-4 was performed with an unintended long interval between LAV priming and the first mVLP booster injection. Low levels of neutralizing antibody induced with cD4-3pm were sustained up to the seventh month after LAV priming when all three macaques responded well to the first mVLP boost (Fig. 4A). The neutralizing antibody titer at the time of challenge in the immunized group was comparable to the plateau level of the control group (Fig. 4B), but only two out of three immunized macaques were protected against a clinical DENV-4 strain, which attained high levels of viremia in the infected control macaques (Fig. 4C). Unexpectedly, a breakthrough infection occurred in a macaque (‘b’) showing the highest level of neutralizing antibody against the challenge serotype in the immunized group (Fig. 4A). None of the immunized macaques in this arm had viremia or viral RNAemia following the challenge (Fig. S3L; Fig. 4D) in contrast to the high levels of viremia and viral RNAemia detected in the control macaques (Fig. 4C and E).

**Fig 5:**
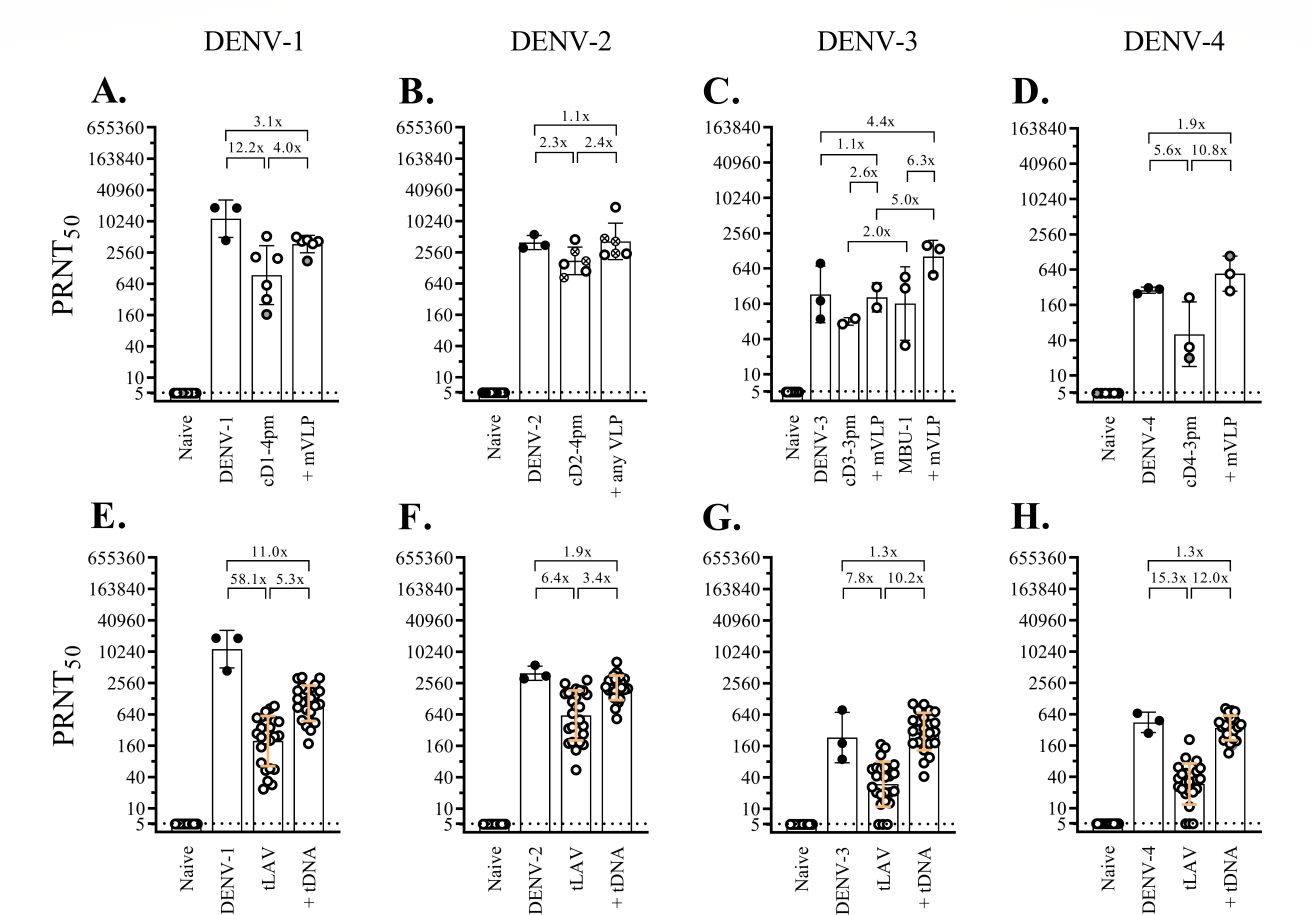
Neutralizing antibody activity 1 month after dengue virus infection or immunization against dengue viruses. The neutralizing antibody titers at baseline (naive), 1 month after infection with wild-type virus (DENV-1, -2, -3, or -4), and 1 or 2 months after immunization with chimeric virus (cD1-4pm, cD2-4pm, cD3-3pm, MBU-1, or cD4-3pm) or a combination of chimeric dengue viruses (tLAV), and 1 month after two successive doses of VLP (+ mVLP or +any VLP) or tetravalent (prM + E)-expressing plasmids (+tDNA) are shown as black circles (infection group) or empty circles (immunization group). (A–D) Groups of two to six macaques were infected with clinical strains of dengue virus or received monovalent heterologous prime-boost immunization. Empty circles with gray shading represent macaques that were subsequently found to be infected with the challenge dengue viruses. The crossed circles in panel (**B**) indicate macaques that received two doses of DENV-2 pVLP. (E–H) Twenty-four macaques were immunized with a combination of chimeric dengue viruses (tLAV) followed by two doses of tetravalent (prM + E)-expressing plasmids (+ tDNA), and PRNT_50_ titers against each dengue serotype were determined and shown separately for all macaques. Prototypic dengue strains were employed in the PRNT, as indicated on the top. The bars and error lines represent geometric means and geometric standard deviations, respectively. The ratio between the geometric means was determined, with the lower value always set as the denominator. The control groups that were infected with DENV-1 (**E**), -2 (**F**), or -3 (**G**) clinical strains were the same as in those panels (**A**), (**B**), and (**C**), respectively. Different groups of macaques were infected with the DENV-4 clinical strain 2-0201-5 (panels D and H).

In the tetravalent heterologous prime-boost immunization, 24 macaques received a priming injection of a mixture of four attenuated chimeric viruses plus two boosting doses of (prM + E)-expressing plasmids via *in vivo* electroporation. One month after the second booster injection, all immunized macaques seroconverted to the four dengue serotypes (Fig. 5E through H). The mean neutralizing antibody titer against DENV-1 was about an order of magnitude lower in immunized macaques than in the DENV-1-infected control macaques (Fig. 5E), whereas the mean titers against other serotypes were in the same range as those detected in wild-type virus-infected macaques (Fig. 5F through H). Four subgroups of six macaques each were then challenged with the same set of clinical dengue strains as in the monovalent approach (Fig. 1F, 2F, 3F, and 4F). Based on the observed minimal changes in the neutralizing antibody titer following the challenge, all macaques in the three subgroups were protected against infection with DENV-1, DENV-2, or DENV-3 clinical strains (Fig. 1F, 2F, and 3F, respectively). Viremia was not detected in these 18 macaques (Fig. S3M through O), and viral RNAemia was minimal during the 2-week post-challenge period (Fig. 1G, 2G, and 3G). In the fourth subgroup, two of the immunized macaques were infected with a DENV-4 clinical strain (Fig. 4F). Viral RNAemia in the second week after the challenge was detected in one infected macaque, although viremia was not detected (Fig. 4I; Fig. S3P). Although the neutralizing antibodies at the time of challenge were within the same order of magnitude among all macaques in this subgroup, two macaques (‘c’ and ‘d’) showed DENV-4 infection (Fig. 4F).

Comparisons of the neutralizing antibody response between male and female macaques were performed in the tetravalent prime-boost immunization arm 60 days after LAV priming and 30 days after the second booster injection (day 118). Despite the large disparity in body weight (Fig. S2A), there was no statistically significant difference in the PRNT_50_ titer between male and female macaques at either time point (Fig. S2B through I).

Overall, the protective efficacy of prime-boost immunization against dengue virus infection was similar between the pooled monovalent arms (18/20 macaques protected, 90%) and the tetravalent arm (22/24 macaques protected, 92%). This result suggested that the different ways in which LAVs were administered, i.e., monovalent vs tetravalent formulations, and the boosting antigens delivered to the immune system, i.e., purified VLP preparations for the exogenous pathway of antigen presentation vs endogenous generation of VLP in plasmid-transfected cells, are unlikely to have a strong impact on the measured efficacy. When the results from the two heterologous prime-boost approaches were combined, the efficacy against dengue virus infection was 90.9% (40/44 macaques protected). When results were segregated according to the infecting serotype, the lowest efficacy of 66.7% (6/9 macaques protected) was observed for macaques challenged with DENV-4.

### Protection against DENV-2 and -3 challenges coincided with unusual anti-EDIII antibody response to prime-boost immunization

**Fig 6:**
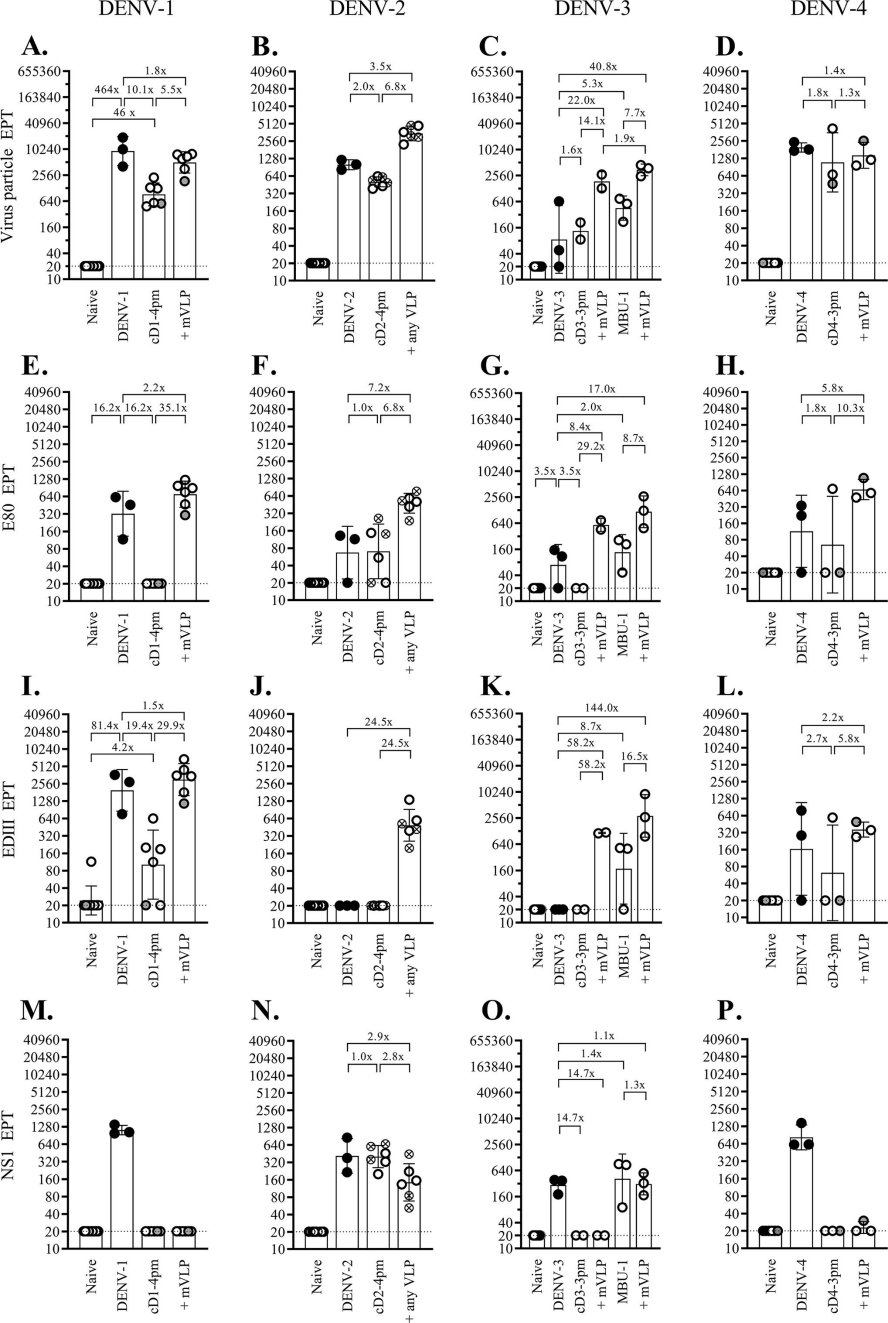
Changes in the binding antibodies recognizing dengue virus particle, E80 and EDIII fragments of the E protein, and the NS1 protein following tetravalent prime-boost immunizations. Twelve macaques that received tetravalent heterologous prime-boost immunizations and subsequently challenged with either DENV-2 or DENV-4 clinical strains were bled at 1 month after receiving the combination of chimeric viruses (tLAV) or boosted with two successive doses of tetravalent (prM + E)-expressing plasmids (+ tDNA). Blood samples were tested by ELISA for dengue virus particles (A–D), E80 fragment (E–H), EDIII fragment (I–L), and NS1 protein (M–P). The viral particles/proteins derived from the four dengue serotypes used in the assays are indicated at the top. Black circles represent macaques challenged with the following clinical dengue strains: 03-0398 (**A, E, I, and M**), 03-0420 (**B, F, J, and N**), 06-129 (**C, G, K, and O**), and 2-0201-5 (**D, H, L, and P**). Empty circles with gray shading represent immunized macaques that were subsequently found to be infected with the challenge dengue viruses. The limit of detection for the first dilution of the sample (1:40) was 20, as indicated by the dotted line. The bars and error lines represent geometric means and geometric standard deviations, respectively. The ratio between the geometric means was determined with the lower value always set as the denominator.

**Fig 7:**
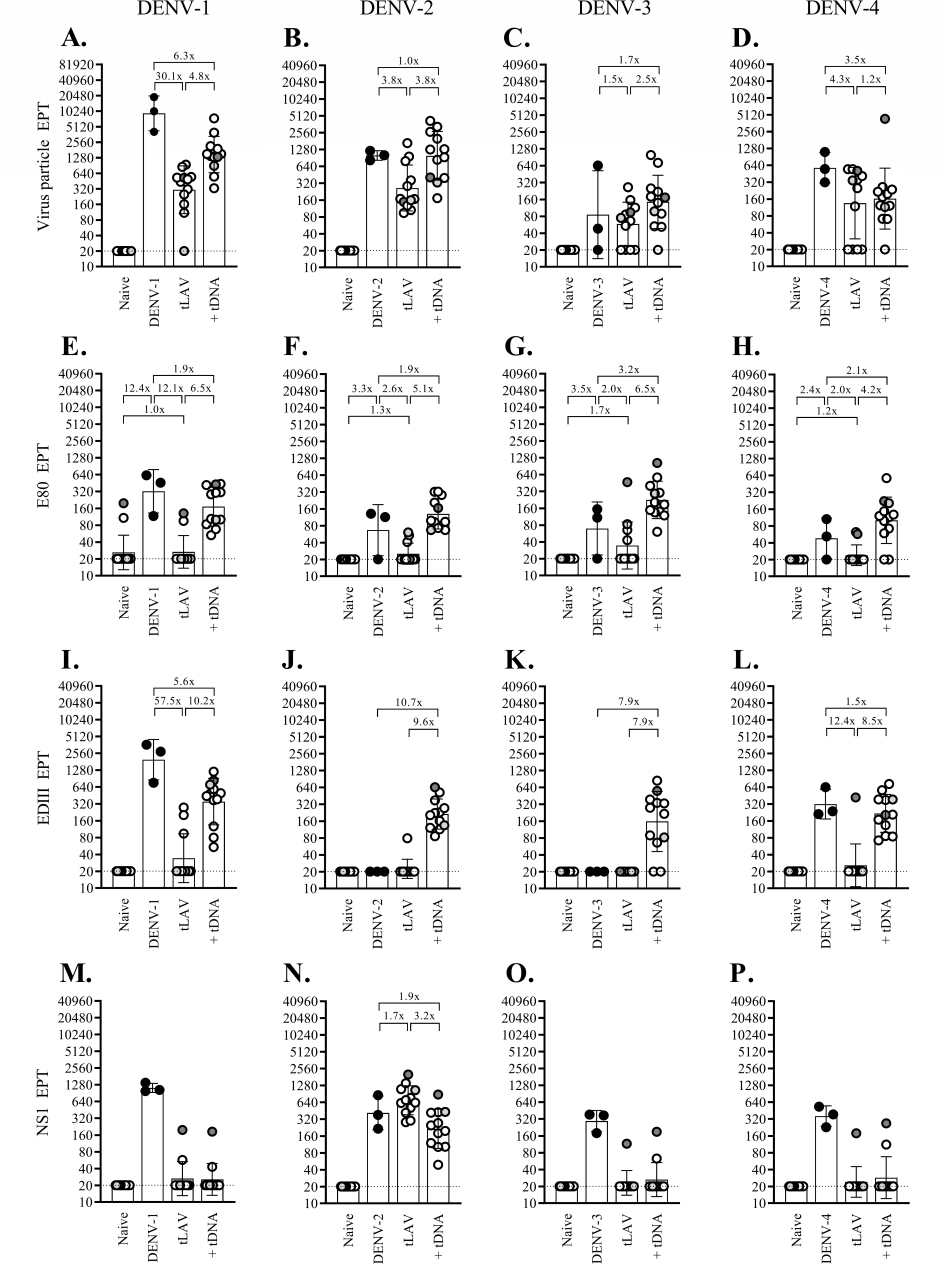
Changes in the binding antibodies recognizing dengue virus particle, E80 and EDIII fragments of the E protein, and the NS1 protein following monovalent prime-boost immunizations. Groups of two to six macaques were infected with recent dengue virus strains or received four separate sets of monovalent heterologous prime-boost immunization. Blood samples taken 1 month after infection or immunization were tested by ELISA against dengue virus particles (A–D), E80 fragment (E–H), EDIII fragment (I–L), and NS1 protein (M–P). Viral particles/proteins derived from the four dengue serotypes used in the assays are indicated at the top. Black circles represent macaques challenged with the following clinical dengue strains: 03-0398 (A, E, I, and M), 03-0420 (B, F, J, and N), 06-129 (C, G, K, and O), and 2-0201-5 (D, H, L, and P). Empty circles with gray shading represent immunized macaques that were subsequently found to be infected with the challenge dengue viruses. Crossed circles in panels (B, F, J, and N) represent macaques boosted with DENV-2 pVLP. The limit of detection for the first dilution of the sample (1:40) was 20, as indicated by the dotted line. The bars and error lines represent geometric means and geometric standard deviations, respectively. The ratio between the geometric means was determined with the lower value always set as the denominator.

It is intriguing that breakthrough infections following live virus challenge in the 44 immunized macaques occurred only with the DENV-1 and DENV-4 challenge strains. To examine the possible underlying mechanisms for this observation, antibodies capable of binding to virus particles and two viral glycoproteins, E and NS1, were assessed in blood samples taken at the time of challenge. Antibodies to prM were not measured as they are generally non-neutralizing and are unlikely to cause infection enhancement in the presence of abundant neutralizing antibodies in the early post-immunization period. Figure 6 shows levels of the antibodies binding to homologous virus particles (A–D), E80 fragments (E–H), EDIII domains (I–L), and NS1 (M–P) separately for the four monovalent arms. Cross-reacting antibodies are shown in Fig. S4 to S7. For the tetravalent immunization approach, binding antibodies were determined in 12 macaques from the two subgroups that were challenged with DENV-2 or DENV-4 strains (Fig. 7).

In both immunization approaches, prime-boost immunization generally raised the mean binding antibody titers to similar (1- to 3.9-fold difference) or markedly higher (greater than or equal to fourfold) levels than those in the control groups (Fig. 6 and 7). In two unusual cases, anti-EDIII antibody response was detected in all, or most, fully immunized macaques in the DENV-2- and DENV-3-monovalent arms (Fig. 7J and K) and the two tetravalent subgroups studied (Fig. 6J and K), but not the wild-type DENV-2- and -3-infected control macaques. These results contrasted with the high anti-EDIII antibody levels in all three wild-type DENV-1-infected control macaques (Fig. 7), as well as five out of six DENV-4-infected control macaques (Fig. 6L and 7L). The paucity of the anti-EDIII response observed in wild-type DENV-2- and -3-infected control macaques suggested that the anti-EDIII antibody might have a minor role in shaping host responses to DENV-2 and -3, and, therefore, the anti-EDIII antibody generated by immunization could have a different impact on the infectivity of DENV-2 and -3 as compared to DENV-1 and -4.

With the exception of the DENV-3-derived MBU-1 strain, all other LAVs employed in this study were (prM + E)-chimeric viruses generated in the genetic background of a prototypic DENV-2 strain. When the titers of anti-NS1 antibodies were determined in macaques after LAV priming using NS1 from all four serotypes, anti-NS1 antibodies reactive to DENV-2 NS1 were detected in macaques primed with cD1-4pm (Fig. S4N), cD2-4pm (Fig. 7N; Fig. S5N), cD3-3pm (Fig. S6N), and cD4-3pm (Fig. S7N) as expected from the presence of the DENV-2 non-structural coding region in these chimeric viruses. Strain MBU-1-primed macaques showed high levels of anti-DENV-3 NS1 antibodies (Fig. 7O; Fig. S6O) and low levels of anti-DENV-1, -2, and -4 NS1 antibodies, which likely reflected cross-reactivities (Fig. S6M, N, and P, respectively).

Anti-NS1 antibodies that reacted with homologous NS1 antigens at the time of challenge with DENV-1 and DENV-4 were present at very low or undetectable levels in the respective monovalent arms (Fig. S4M and S7P, respectively), indicating that type-specific anti-NS1 antibodies are not required for protection against DENV-1 and DENV-4 challenges.

### A structural basis for antibody cross-reactivity

Examination of the binding activity of the anti-EDIII antibodies in DENV-1- and DENV-4-infected control macaques using isolated EDIII domains of the four dengue serotypes in ELISA revealed minimal binding of macaque anti-EDIII antibodies to heterologous EDIII domains (Fig. S4I through L, S7I through L, and S8C). Similar findings were found for the anti-E80 antibody responses in control macaques (Fig. S4E through H, S5E through H, and S7E through H). The predominantly monospecific anti-E80 and anti-EDIII antibodies were in contrast to those binding to virus particles, which exhibited more extensive cross-reactive binding activities (Fig. S4A through D, S5A through D, S7A through D, and S8A). These results indicate that antibodies recognizing the isolated EDIII domain and E80 fragment, which are devoid of quaternary structure-dependent epitopes, tend to be type-specific, whereas those recognizing multimeric structures, including the prM-E dimer, E-E dimer, and morphological hexameric raft present on the surface of dengue virus particles are likely to be cross-reactive.

### Two distinct mechanisms account for dengue virus breakthrough infection in immunized cynomolgus macaques

Among the four immunized macaques that developed infections following live viral challenge, one in the monovalent DENV-1 arm (‘a’) and the other in the tetravalent DENV-4-challenge subgroup (‘c’) had the lowest levels of neutralizing antibodies at the time of challenge when compared to protected macaques in the same groups (Fig. 1A and 4F). Macaque ‘a’ also had the lowest levels of DENV-1 particle-binding, E80-binding, and EDIII-binding antibodies (Fig. 6A, E, and I), which is consistent with the failure to mount an adequate antibody response required for protection. In contrast, two other macaques with breakthrough infection—‘b’ in the monovalent DENV-4 arm and ‘d’ in the tetravalent DENV-4 subgroup—exhibited relatively high neutralizing antibody activities against DENV-4 (Fig. 4A and F). Macaques ‘b’ and ‘d’ also had high titers of virus particle-binding antibodies (Fig. 6D and 7D). These results indicate distinct mechanisms by which breakthrough infection occurs in immunized macaques.

**Fig 8:**
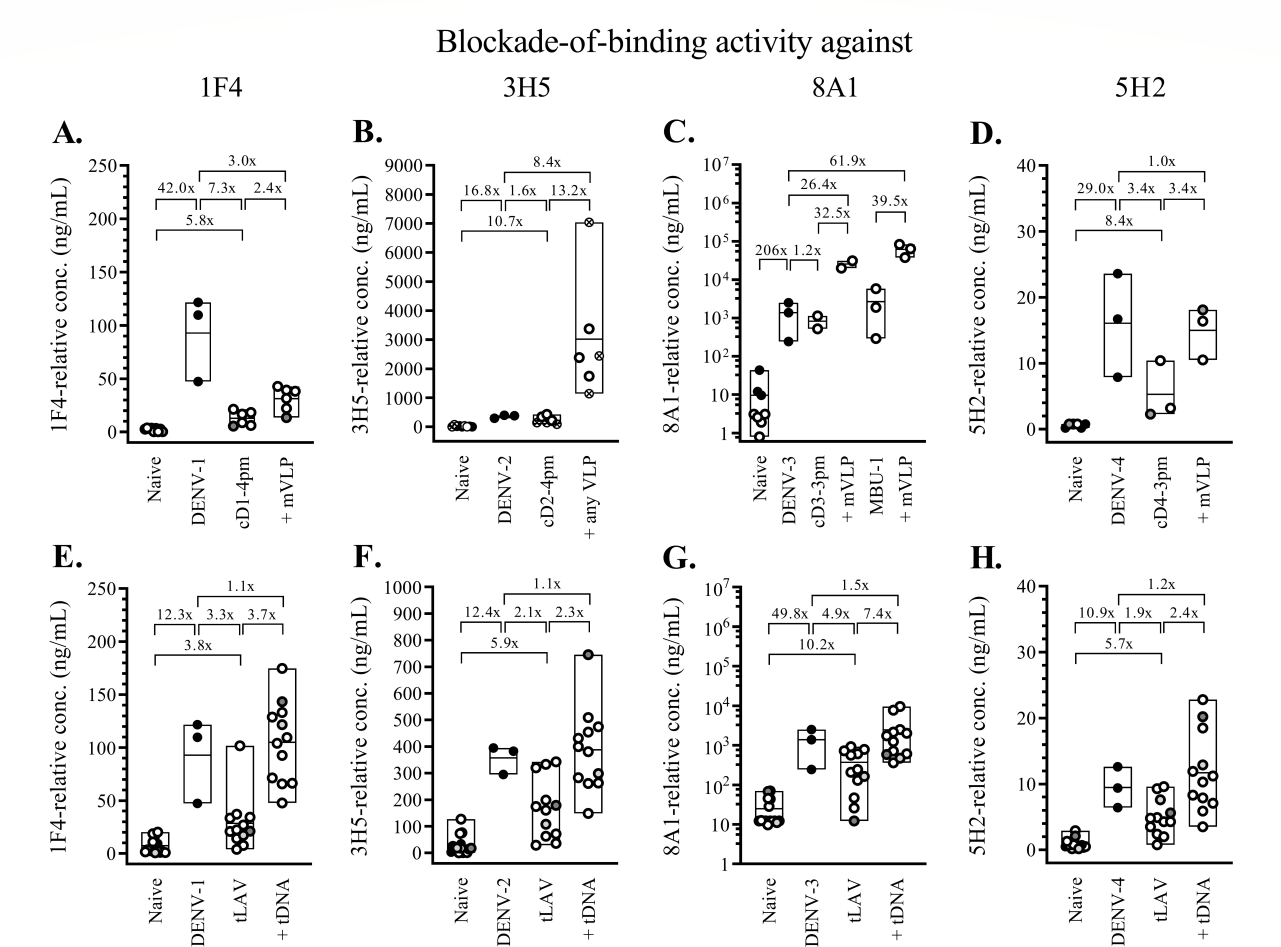
Blockade-of-binding activity 1 month after dengue virus infection or immunization against dengue viruses. The blockade-of-binding activity at baseline (naive), 1 month after infection with clinical dengue isolates (DENV-1, -2, -3, or -4), and 1 month after immunization with a chimeric dengue virus (cD1-4pm, cD2-4pm, cD3-3pm, MBU-1, or cD4-3pm) or a combination of chimeric viruses (tLAV), and 1 month after two successive doses of VLP (+mVLP or +any VLP) or tetravalent (prM + E)-expressing plasmids (+tDNA) are shown as black circles (after infection) or empty circles (after immunization). (A–D) Blockade-of-binding activities for groups of two to six macaques infected with clinical dengue virus strains or that had received DENV-1 (**A**), DENV-2 (**B**), DENV-3 (**C**), or DENV-4 (**D**)-related monovalent heterologous prime-boost immunization. (E–H) Blockade-of-binding activities for 12 macaques that had received tetravalent heterologous prime-boost immunizations and were subsequently challenged with either DENV-2 or DENV-4 clinical strains. Blood samples were employed in the blockade-of-binding ELISA using the following pairs of serotype-specific, HRP-conjugated antibodies and the corresponding dengue viruses: 1F4-DENV-1 (**A and E**), 3H5-DENV-2 (**B and F**), 8A1-DENV-3 (**C and G**), and 5H2-DENV-4 (**D and H**). The minimum and maximum values and geometric means of each group are shown as box plots. The ratio between the geometric means was determined with the lower value always set as the denominator. Different groups of macaques were infected with a DENV-4 clinical strain in panels (**D) and (H**). Empty circles with gray shading represent macaques that were subsequently found to be infected with the challenge dengue viruses. The crossed circles in panel (**B**) represent macaques that were boosted with DENV-2 pVLP.

To examine whether macaques with breakthrough infections also differed from their protected counterparts in their ability to generate antibodies recognizing selected type-specific epitopes, the blockade-of-binding ELISA was employed (Fig. 8). A set of strongly neutralizing, type-specific monoclonal antibodies (1F4, 3H5, 8A1, and 5H2), which represent type-specific antibodies for DENV-1, DENV-2, DENV-3, and DENV-4, respectively, was shown with this assay to measure type-specific binding antibody activities with moderate or strong correlations with the corresponding neutralizing antibody titers in macaques (33). In agreement with the data from particle-binding experiments, the lowest relative level of 1F4-blocking activity at the time of challenge was detected in macaque ‘a’ (Fig. 8A), whereas the highest relative level of 5H2-blocking activity occurred in macaque ‘b’ (Fig. 8D). Macaque ‘d’ had the highest 3H5-blocking activity (Fig. 8F) and the second-highest level of 1F4-blocking (Fig. 8E) and 5H2-blocking (Fig. 8H) activities. These results precluded a possibility that the two macaques with high neutralizing antibody responses to DENV-4 were infected with the challenge virus because they were unable to generate antibodies that targeted type-specific epitopes.

**Fig 9:**
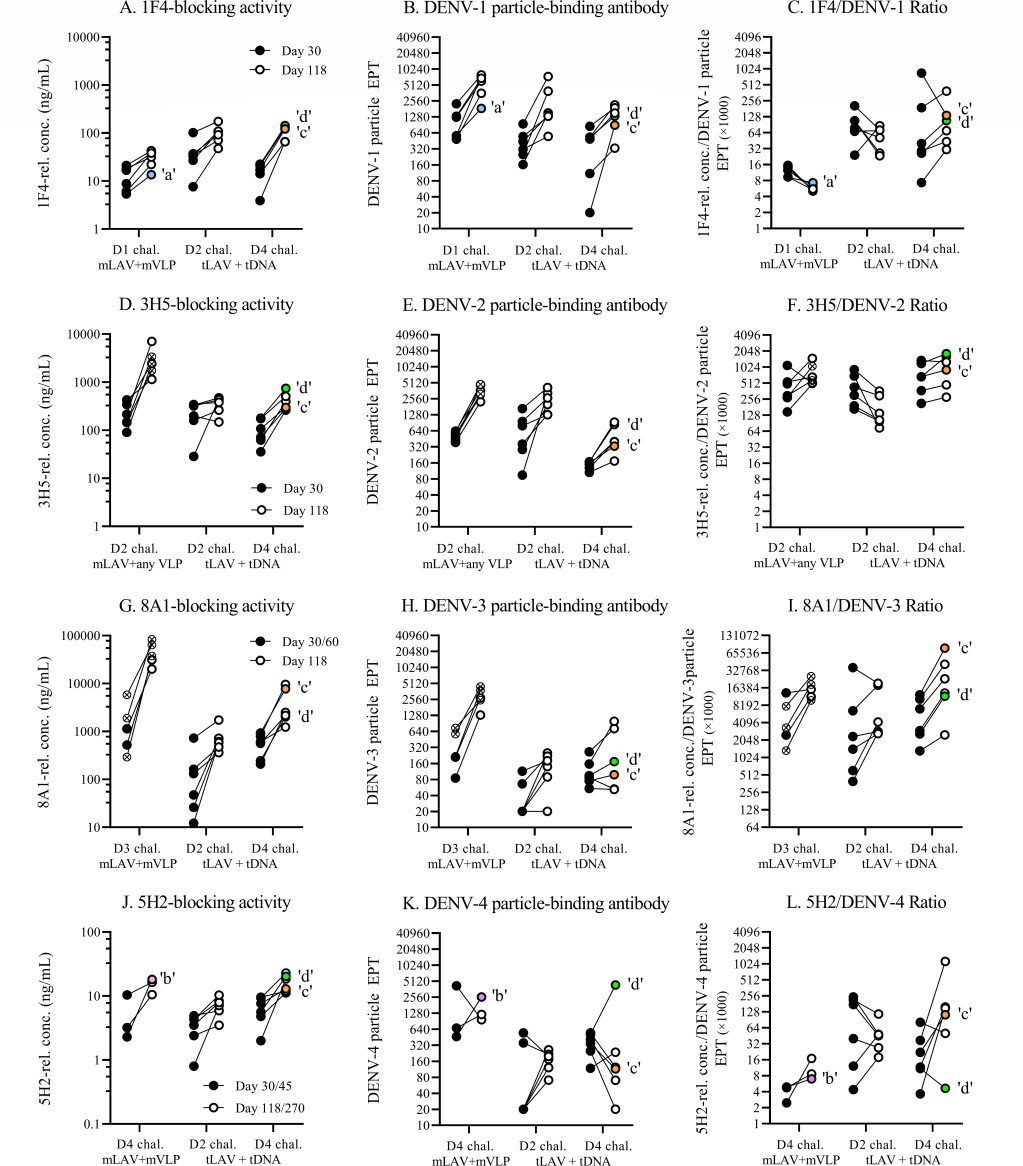
Changes in the blockade-of-binding activity, virus particle-binding antibody, and blocking/binding ratio during prime-boost immunizations. The relationships between the blockade-of-binding activity, particle-binding antibody, and their ratio are shown at the day of peak PRNT titer following the priming LAV injection (day 30, day 45, or day 60; black solid circles) and 1 month after the second boosting injection (day 118 or day 270, empty circles) for the four monovalent prime-boost immunization groups and two tetravalent prime-boost immunization groups. Empty circles with colored shading represent macaques that were subsequently found to be infected with the challenge dengue viruses: macaque ‘a’ [light blue circles in panels (A)–(C), D1 challenge mLAV + mVLP], macaque ‘b’ [purple circle in panels (J)–(L), D4 challenge mLAV + mVLP], macaque ‘c’ [orange circle in panels (A)–(L), D4 challenge + tDNA], macaque ‘d’ [green circle in panels (A)–(L), D4 challenge + tDNA]. Crossed circles in the left section of panels (D)–(F) represent DENV-2 LAV-primed macaques boosted with pVLP. Crossed circles in the left section of panels (G)–(I) denote macaques primed with DENV-3 LAV strain MBU-1.

A caveat in using the neutralizing antibody titer, which is determined from the endpoint of serial dilutions of sample causing a designated level of plaque or focus reduction, is that it does not necessarily reflect the *in vivo* situation where a mixture of antibodies with different properties, i.e., concentration, affinity, and interfering capability, exert their activities in combination. As an alternative way to understand the interaction between dengue virus and antibody, the ratio of the antibody titer recognizing a particular epitope to the total particle-binding activity may better represent the *in vivo* biological properties of interest. The ratio between the type-specific epitope-blocking activity and the virus particle-binding antibody titer was determined for macaque samples after LAV priming (days 30, 45, or 60, depending on the time of peak PRNT titer) and just before the time of challenge (days 118 or 270) (Fig. 9). Macaques ‘b’ and ‘d’ displayed the lowest 5H2/DENV-4 ratios compared to the protected macaques in the same groups (Fig. 9L). Excessive production of DENV-4 particle-binding antibody (Fig. 9K), but no reduction in 5H2-blocking activity (Fig. 9J) in response to booster immunization are responsible for the peculiarly low 5H2/DENV-4 ratios in these two macaques. The response of macaque ‘d’ to DENV-4 particle boost was unique, as the 1F4/DENV-1 ratio (Fig. 9C), 3H5/DENV-2 ratio (Fig. 9F), and 8A1/DENV-3 ratio (Fig. 9I) found in this macaque lie within the same ranges as those in other tetravalently primed-boosted macaques. Similar low blocking activity/DENV-4 particle-binding ratios were observed in macaques ‘b’ and ‘d’ with two HRP-conjugated antibodies (513 and EDE-1 C10) recognizing dengue virus common epitopes (Fig. S9).

## DISCUSSION

The high overall efficacy of LAV-primed/non-infectious particle-boosted immunization in preventing dengue virus infection observed in this study shows how improvements can be made to dengue vaccines. The LAVs used in this study were very similar to TAK-003. While the backbone of TAK-003 chimeric vaccine strains was derived wholly from DENV-2 strain 16681-PDK 53 (35), our LAVs were constructed based on strain 16681 with only the three genetically introduced attenuating mutations (5′ UTR C57T, NS1 Gly53Asp, and NS3 Glu250Val) of 16681-PDK 53. The sequences of the chimeric prM-E coding region employed in this study were obtained from dengue virus-infected Thai patients during 2003–2006, representing more recent strains as compared to the antigens used in Dengvaxia, TAK-003, and TV003, which were derived from dengue viruses circulating during 1944–1988. A comparison with our previous single-dose monovalent LAV immunization study (31), wherein none of the 12 cD1-3pm- or cD1-4pm-immunized rhesus macaques was protected against the same DENV-1 challenge virus, suggests that, at least in the DENV-1 challenge model, the improved protection found in this study is a consequence of the booster immunization. Using a criterion of a greater than or equal to fourfold increase in neutralizing antibody activity after challenge, the high efficacy achieved in this study contrasts with previous studies that employed one or two doses of tetravalent LAVs (25, 26) or other heterologous prime-boost strategies (61, 62). Whether this level of efficacy, as shown here in macaques, could be realized in humans, particularly among dengue virus-naïve young children, needs to be addressed in clinical trials.

A remarkable finding in this study was the induction of full seroconversion in macaques at the completion of heterologous prime-boost immunization (Fig. 5). This represents an improvement over the homologous LAV prime-boost regimen, such as that of the two-dose TAK-003 vaccine, in which a previous study showed that 5 out of 32 macaques did not have neutralizing antibodies to DENV-4 by the end of immunization (26). More pertinently, an advantage of heterologous booster immunization is its ability to stimulate neutralizing antibodies in all four macaques that failed to mount neutralizing antibody responses against DENV-3 or -4 in the 2-month period following tLAV priming (Fig. 5G and H). In comparison, a second dose of TAK-003 was shown to induce seroconversion in only four out of nine macaques that failed to generate neutralizing antibodies against DENV-4 after the first LAV dose (26).

While several monoclonal antibodies that recognize type-specific epitopes in the EDIII domain are potently neutralizing (63), anti-EDIII antibodies are present in primary and secondary DENV-infected human sera at low levels and play a minor role in DENV neutralization *in vitro* (48, 64, 65). However, all monovalently immunized macaques in this study were able to mount high levels of type-specific anti-EDIII antibodies (Fig. 6I through L), and the majority of macaques in the tetravalent approach displayed broad EDIII-binding antibody activities (Fig. 7I through L). The importance of booster immunization is supported by the observation that VLP stimulated the production of anti-EDIII antibodies in all 13 macaques that failed to do so after receiving monovalent LAV injections (Fig. 6I through L). Tetravalent DNA boosters also provide effective stimuli for the generation of EDIII-binding antibodies in all or, in the case of DENV-3 EDIII, the majority of macaques that were unable to generate anti-EDIII antibody following tetravalent LAV priming (Fig. 7I through L). More importantly, EDIII-binding antibodies were detected simultaneously with those recognizing the EDIII-associated type-specific epitopes of DENV-2 and DENV-3 in related monovalently immunized macaques, as well as in the majority of tetravalently immunized macaques (Fig. 8). This finding is in contrast to the shift in EDIII-binding antibodies from those recognizing type-specific epitopes toward those of cross-reactive epitopes that have been observed in humans following secondary dengue virus infections (48). The persistence of antibodies recognizing EDIII-associated type-specific epitopes may contribute to the full protection observed in DENV-2- and -3-challenged macaques in this study. As noted earlier, the absence of EDIII-binding antibodies in macaques infected with wild-type DENV-2 and -3 in this study and the failure to detect specific anti-EDIII antibody responses in individuals infected with non-DENV-1 serotypes (66) raises the possibility that anti-EDIII antibodies play a minor role in shaping host responses to DENV-2 and -3.

Studies performed in seronegative Dengvaxia and TV003 recipients with breakthrough infections have shown that homotypic neutralizing antibodies are a better correlate of protection than the total level of neutralizing antibodies (17, 67). However, whereas 76% of TV003 recipients was able to mount homotypic neutralizing antibodies against DENV-2 (PRNT_50_ titer of ≥20), only 57% could be protected (17, 27). This discrepancy suggests that other factor(s) may be involved in determining *in vivo* protection against dengue. Breakthrough infections in the presence of high levels of neutralizing antibodies to the infecting serotype have been observed in children and infants in natural settings (68, 69). Whereas low levels (PRNT_50_ < 60) of pre-existing neutralizing antibodies to the infecting serotypes accounted for about half (17/31) of breakthrough infections in Thai children in a longitudinal observational study, pre-existing antibodies in the high PRNT_50_ ranges of 121–1,848 and 5,291–11,682 against the infecting serotypes 1 and 4, respectively, were found in up to 29% (9/31) of volunteers (68). In this study, two macaques experienced DENV-4 breakthrough infection despite relatively high levels of pre-existing neutralizing and particle-binding antibodies. In both macaques, the 5H2-blocking/DENV-4-binding antibody ratio was at the lowest levels as compared to others in the corresponding groups, suggesting that the presence of other viral particle-binding antibodies may compromise the virus-neutralization ability of antibodies directed against this neutralization-associated epitope to a greater extent in these two macaques than in others. Almost identical findings were obtained with antibodies recognizing the two common epitopes, 513 and EDE-1 C10 (Fig. S9), but these may be of minor significance as the corresponding blockade-of-binding activities do not correlate with *in vitro* virus neutralization as strongly as the 5H2 epitope-blocking activity (31).

This study had several limitations. Rigorous statistical analysis was not performed because too few macaques were employed in the control groups and monovalent prime-boost subgroups. While a short 1-month interval between immunization and challenge was used, as had been used previously in the TAK-003 challenge study (26), it did not permit the assessment of long-term efficacy or risks associated with waning immunity (70). Neutralizing antibodies persist in both macaques and humans following dengue virus infection for years (24, 71); hence, it is likely that, with the elevated levels of neutralizing antibodies observed following the prime-boost immunization in this study, the protective efficacy would not change drastically if challenges were performed after a longer interval. The low level of viremia in non-immunized macaques infected with strain 06-129 may imply a reduced virulence of this virus, although productive infection could be readily achieved with this strain in other macaques, and dose escalation did not extend the duration of viremia or increase the proportion of viremic macaques.

### Future perspectives

The high overall efficacy of heterologous LAV-primed/non-infectious particle-boosted immunization in macaques justifies further study in humans, as this approach may be beneficial in the prevention of dengue in young seronegative recipients or asymptomatic infection. The inclusion of females and the finding of comparable levels of neutralizing antibodies in both sexes enhance the relevance of our study and should be helpful in the planning of future clinical trials. Additional studies on other designs of boosting antigens or lower complexities may help broaden the choices of potential boosting agents. This may include whole or truncated E protein or selected E domain, which may represent a better immunogen for the stimulation of serotype-specific antibodies, by virtue of their lack of the quaternary structure-dependent epitopes. The findings that a number of “high responder” macaques are not protected from live virus challenge raise a question regarding the use of serotype-specific “cutoff” levels of neutralizing antibodies as *in vitro* correlates of the protective immune response against dengue.

## AFTER EPUB

*[This article was published on 17 October 2023 with Fig. 6 missing and Fig. 7 appearing in its place. The figure was updated in the current version, posted on 31 October 2023.]*

## SUPPLEMENTAL MATERIAL

Supplemental file jvi.00963-23-s0001.docx Table S1 and Fig. S1 to S9.

ASM does not own the copyrights to Supplemental Material that may be linked to, or accessed through, an article. The authors have granted ASM a non-exclusive, world-wide license to publish the Supplemental Material files. Please contact the corresponding author directly for reuse.
