# Supplementary material for: Heterologous prime-boost immunization induces protection against dengue virus infection in cynomolgus macaques
Source: J Virol. 2023 Oct 17;97(11):e00963-23. doi: 10.1128/jvi.00963-23 (PMC10688363; doi:10.1128/jvi.00963-23)
Supplement: Supplemental file — Table S1 and Fig. S1 to S9. [file jvi.00963-23-s0001.docx]

**Supplementary Materials**

Table S1 Amino acid sequences of mature dengue VLP in this study in FASTA format

1. DENV-1 VLP

SVALAPAVGLGLETRTETWMSSEGAWKQIQRVETWALRHPGFTVIALFLAHAIGTSITQKGIIFILLMLVTPSMAMRCVGIGSRDFVEGLSGATWVDVVLENGSCVTTMAKDKPTLDIELLKTEVTNPAVLRKLCIEAKISNTTTDSRCPTQGEATLVEEQDANFVCRRTFVDRGWGNGCGLFGKGSLLTCAKFKCVTKLEGKIVQYENLKYSVIVTVHTGDQNQVGNESTEHGTTATITPQAPTTEIQLTDYGALTLDCFPLTGLDFNEMVLLTMKEKSWLVNKQWFLDLPLPWTSGASTSQETWNRQDLLVTFKTAHAKKQEVVVLGSQEGAMATALTGATEIQTSGTTTIFAGALKCRLKMDKLTLKGMSYVMCTGSFKLEKEVAETQAGTVLVQIKYEGTDAPCKIPFSTQDEKGVTQNGRLITANPIVTDKEKPVNIEAEPPFGESYIVIGAGEKALKLSWFKKGSTLGKAFSTTLKGAQRLAALGDTAWDFGSIGGVFNSIGKAVHQVFGGAFRTLFGGMSWITQGLMGALLLWMGVNARDRSIALAFLATGGVLVFLATNVHA

1. DENV-2 VLP

SVALVPAVGMGLETRTETWMSSEGAWKHAQRIETWILRHPGFTIMAAILAYTIGTTHFQRVLIFILLTAVAPSMTMRCIGISNRDFVEGVSGGSWVDIVLENGSCVTTMAKNKPTLDFELIKTEAKQPATLRKYCIEAKLTNTTTESRCPTQGEPSLKEEQDKRFVCKHSMVDRGWGNGCGLFGKGGIVTCAMFTCKKNMEGKIVQPENLEYTIVVTPHSGEENAVGNDTGKHGKEIKVTPQSSITEAELTGYGTVTMECFPLTGLDFNEMVLLQMENKAWLVNRQWFLDLPLPWLPGADKQESNWIQKETLVTFKSPHAKKQDVVVLGSQEGAMATALTGATEIQMSSGNLLFTGALKCRLRMDKLQLKGMSYSMCTGKFKVVKEIAETQAGTIVIQVQYEGDGSPCKIPFEIMDLEKRYVLGRLITVNPIVTEKDSPVNIEAEPPFGDSYIIIGVEPGQLKLNWFKKGSTLGKAFSTTLKGAQRLAALGDTAWDFGSIGGVFNSIGKAVHQVFGGAFRTLFGGMSWITQGLMGALLLWMGVNARDRSIALAFLATGGVLVFLATNVHA

1. DENV-3 VLP

SVALAPAVGMGLDTRTQTWMSAEGAWRQVEKVETWALRHPGFTILALFLAHYIGTSLTQKVVIFILLMLVTPSMTMRCVGVGNRDFVEGLSGATWVDVVLENGGCVTTMAKNKPTLDIELQKTEATQLATLRKLCIEGKITNITTDSRCPTQGEAALPEEQDQNYVCKHTYVDRGWGNGCGLFGKGSLVTCAKFQCLEPIEGKVVQYENLKYTVIITVHTGDQNQVGNETQGVTVEITPQASTTEAILPEYGTLGLECFPLTGLDFNEMILLTMKNKAWMVNRQWFFDLPLPWTSGATTETPTWNRKELLVTFKSAHAKKQEVVVLGSQEGAMATALTGATEIQNSGGTSIFAGALKCRLKMDKLELKGMSYAMCTNTFVLKKEVSETQAGTILIQVEYKGEDVPCKIPFSTEDGQGKAHNGRLITANPVVTKKEEPVNIEAEPPFGESNIVIGIGDNALKINWYKKGSTLGKAFSTTLKGAQRLAALGDTAWDFGSIGGVFNSIGKAVHQVFGGAFRTLFGGMSWITQGLMGALLLWMGVNARDRSIALAFLATGGVLVFLATNVHA

1. DENV-4 VLP

SVALTPASGMGLETRAETWMSSEGAWKHAQRVESWILRNPGFALLAGFMAYMIGQTGIQRTVFFVLMMLVAPSYGMRCVGVGNRDFVEGVSGGAWVDLVLENGGCVTTMAQGKPTLDFELTKTTAKEVALLRTYCIEASISNITTATRCPTQGEPYLKEEQDQQYICRRDVVDRGWGNGCGLFGKGGVVTCAKFSCSGKITGNLVQIENLEYTVVVTVHNGDTNAVGNDTSNHGVTAMITPRSPSVEVKLPDYGELTLDCFPLSGIDFNEMILMKMKKKTWLVNKQWFLDLPLPWTAGADTSEVHWNYKERMVTFKSPHAKRQDVTVLGSQEGAMASALAGATEVDSGDGNHMFAGALKCKVRMEKLRIKGMSYTMCSGKFSIDKEMAETQAGTTVVQVKYEGAGAPCKVPIEIRDVNKEKVVGRIISSTPLAENTNSVTNIELEPPFGDSYIVIGVGNSALTLHWFRKGSTLGKAFSTTLKGAQRLAALGDTAWDFGSIGGVFNSIGKAVHQVFGGAFRTLFGGMSWITQGLMGALLLWMGVNARDRSIALAFLATGGVLVFLATNVHA


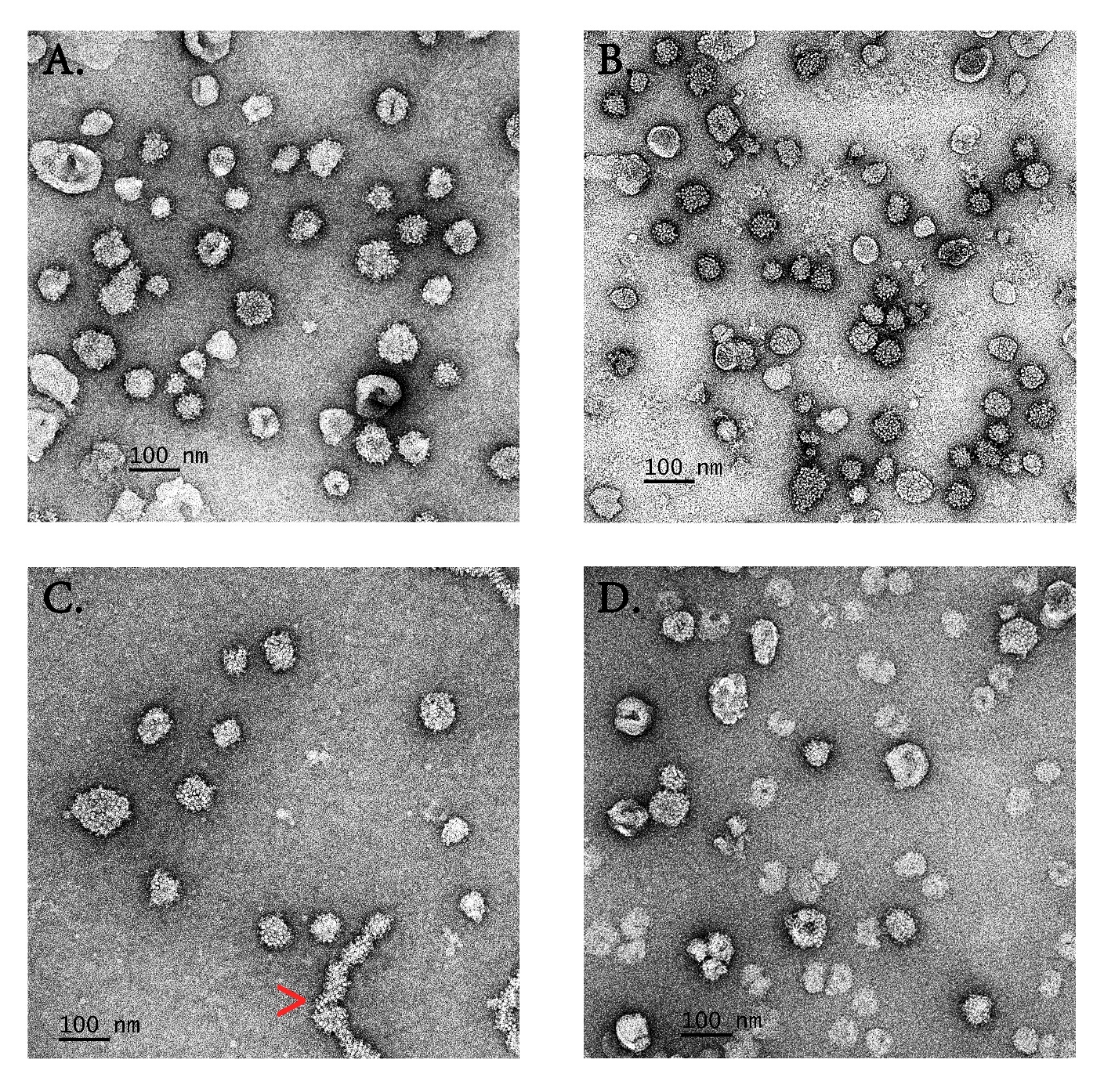


**Fig. S1** Electron micrographs of negatively stained mVLP preparations. Virus-like particles were concentrated from culture media of stably (M+E)-transfected C6/36 cells, purified by successive centrifugations in sucrose gradients, negatively stained with uranyl acetate, and visualized using a transmission electron microscope. Micrographs are arranged according to the corresponding serotypes: **A.**, DENV-1; **B.**, DENV-2; **C.**, DENV-3; and **D.**, DENV-4. Note the difference in size, which is expected of the coreless viral-like particles, and the deformity of shape that may be artifacts from sample purification and preparation for electron microscopy. An elongated particle in panel **C.** (red arrowhead) is reminiscent of club-shaped particles previously described for DENV-3 (60).


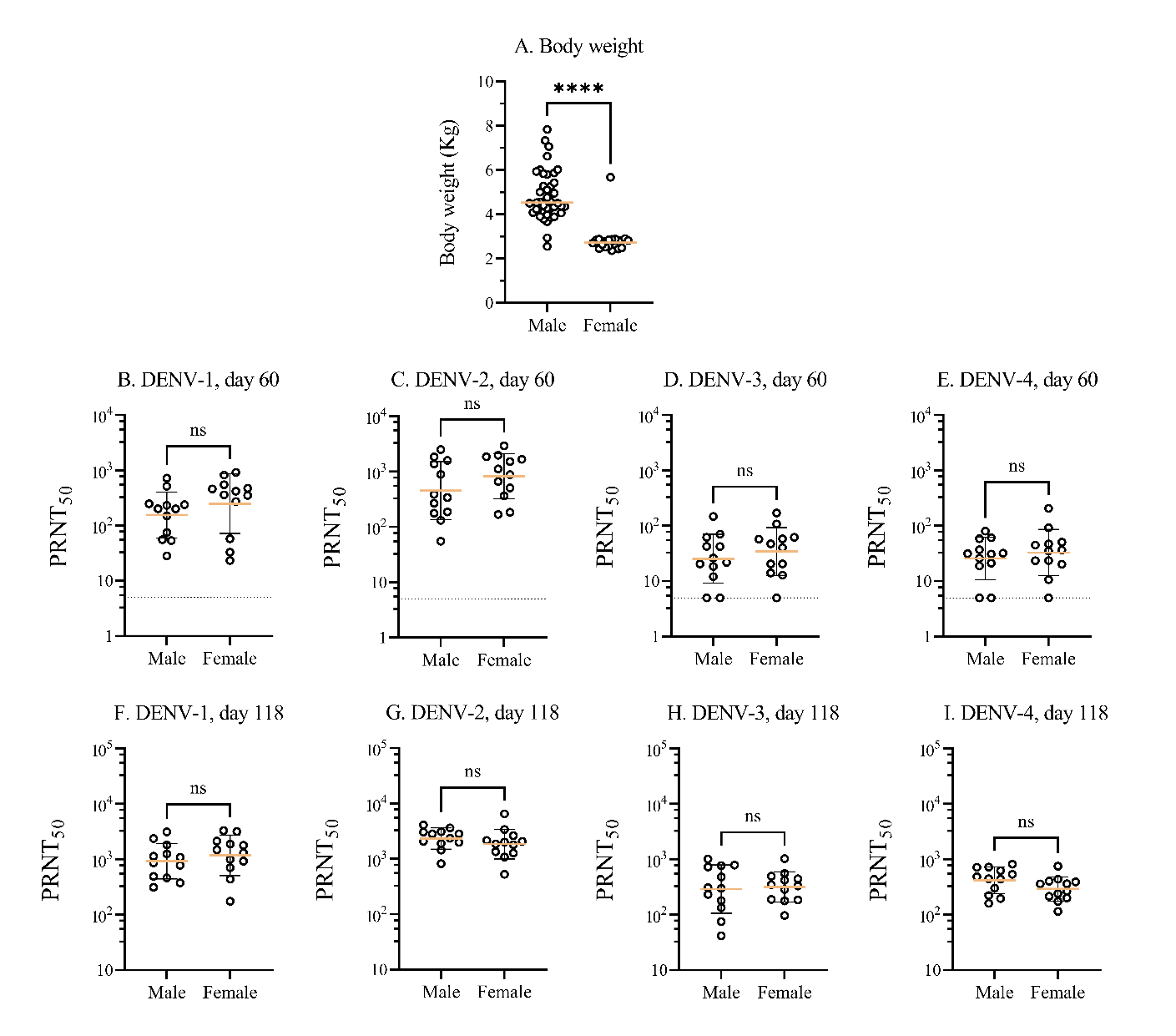


**Fig. S2** Comparisons of body weight and neutralizing antibody titer between male and female cynomolgus macaques. **A.** Body weight at enrollment was compared between male and female macaques. Bar represents the median. **B.–E.** Neutralizing antibody titer (PRNT_50_) for male and female macaques 60 days after tLAV priming. **F.–I.** Neutralizing antibody titer (PRNT_50_) for male and female macaques 30 days after the second DNA boosting dose. The bars and error lines represent geometric means and geometric standard deviations, respectively. The limit of detection for the neutralizing antibody at the first dilution of blood sample (1:10) was 5, as indicated by the dotted line.


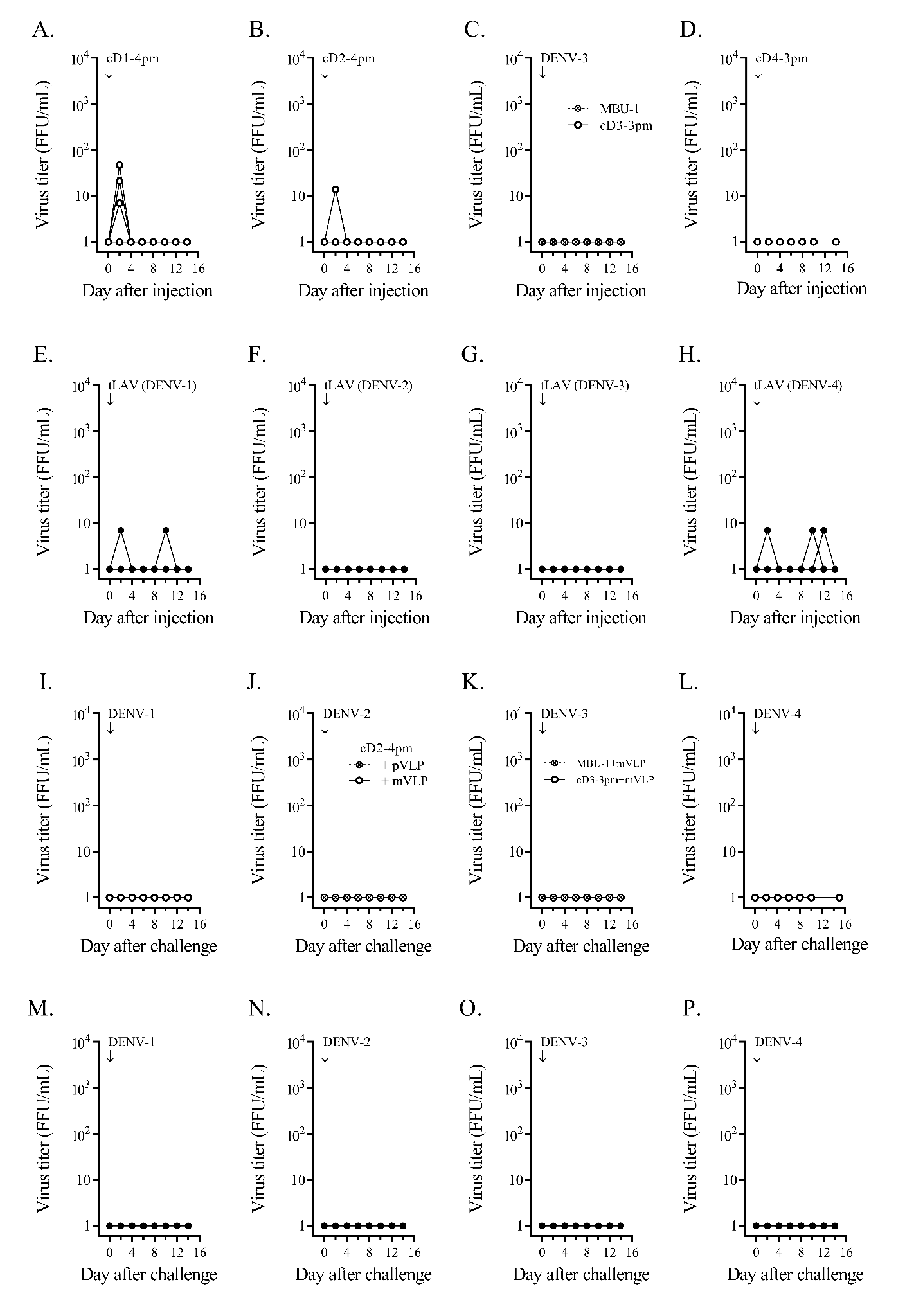


**Fig. S3** Viremia in macaques following priming injection and challenge. **A–D.** Viremia in macaques that were immunized with the priming injection of cD1-4pm (**A**), cD2-4pm (**B**), MBU-1 or cD3-3pm (**C**), and cD4-3pm (**D**) **E– H.** Viremia in 24 macaques that were immunized with the priming tetravalent LAV injection. Four subgroups of six macaques each are shown according to the serotype of clinical dengue strains employed for subsequent challenge: DENV-1, (**E**); DENV-2, (**F**); DENV-3, (**G**) and DENV-4, (**H**). **I****–L.** Viremia in macaques that were challenged with clinical dengue strains: DENV-1 (**I**), DENV-2 (**J**), DENV-3 (**K**) and DENV-4 (**L**) following the priming injection of monovalent LAV and booster injections with VLP, corresponding to panels **A**, **B**, **C,** and **D**, respectively. **M–P.** Viremia in macaques that were challenged with clinical dengue strains: DENV-1, (**M**); DENV-2, (**N**); DENV-3, (**O**) and DENV-4, (**P**) following the priming injection of LAV and booster injections with tetravalent (prM+E)-expressing plasmid DNA corresponding to panels **E**, **F**, **G**, and **H**, respectively. The limit of detection of viremia was 1 FFU/mL, as indicated by the black line.


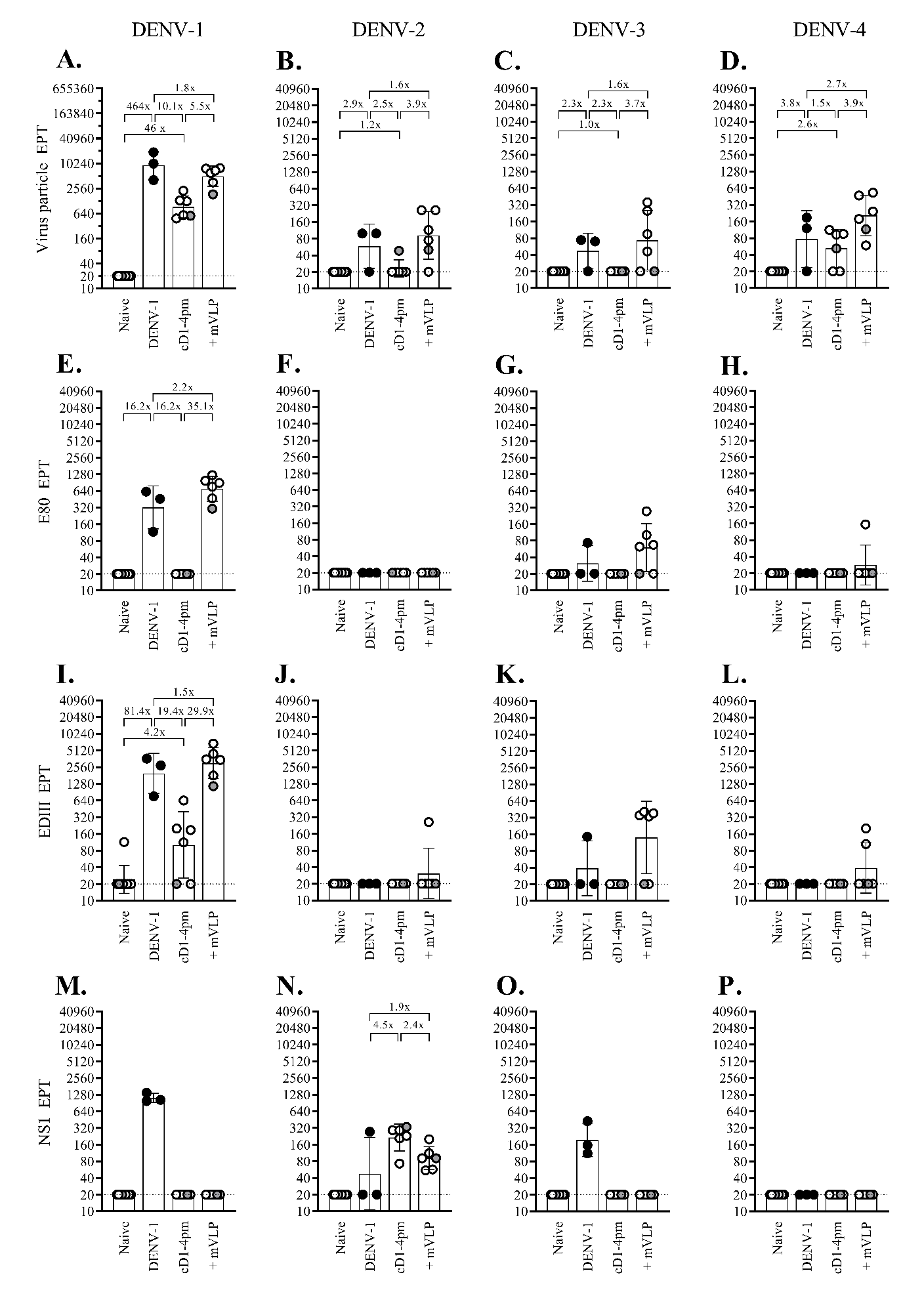


**Fig. S4** Changes in cross-reactive binding antibodies recognizing dengue virus particle, E80 and EDIII fragments of the E protein, and the NS1 protein following DENV-1 infection and related monovalent prime-boost immunization. Groups of three and six macaques were infected with clinical dengue strain 03-0398 (DENV-1) and received monovalent heterologous prime-boost immunization (cD1-4pm) followed by two doses of DENV-1 mVLP (+ mVLP). Blood samples taken one month after infection or immunization were tested by ELISA for dengue virus particles (**A**–**D**), E80 fragment (**E**–**H**), EDIII fragment (**I–L**), and NS1 protein (**M–P**). Viral particle/proteins derived from each of the four dengue serotypes are indicated at the top. Black circles represented non-immunized control macaques infected with strain 03-0398. Empty circles with gray shading represent data from an immunized macaque that was subsequently found to be infected with the challenge dengue virus (macaque `a´). The limit of detection at the first dilution of sample (1:40) was 20, as indicated by the dotted line. The bars and error lines represent geometric means and geometric standard deviations, respectively. The ratios of geometric means were determined with the lower value always set as the denominator. EPT, endpoint titer.


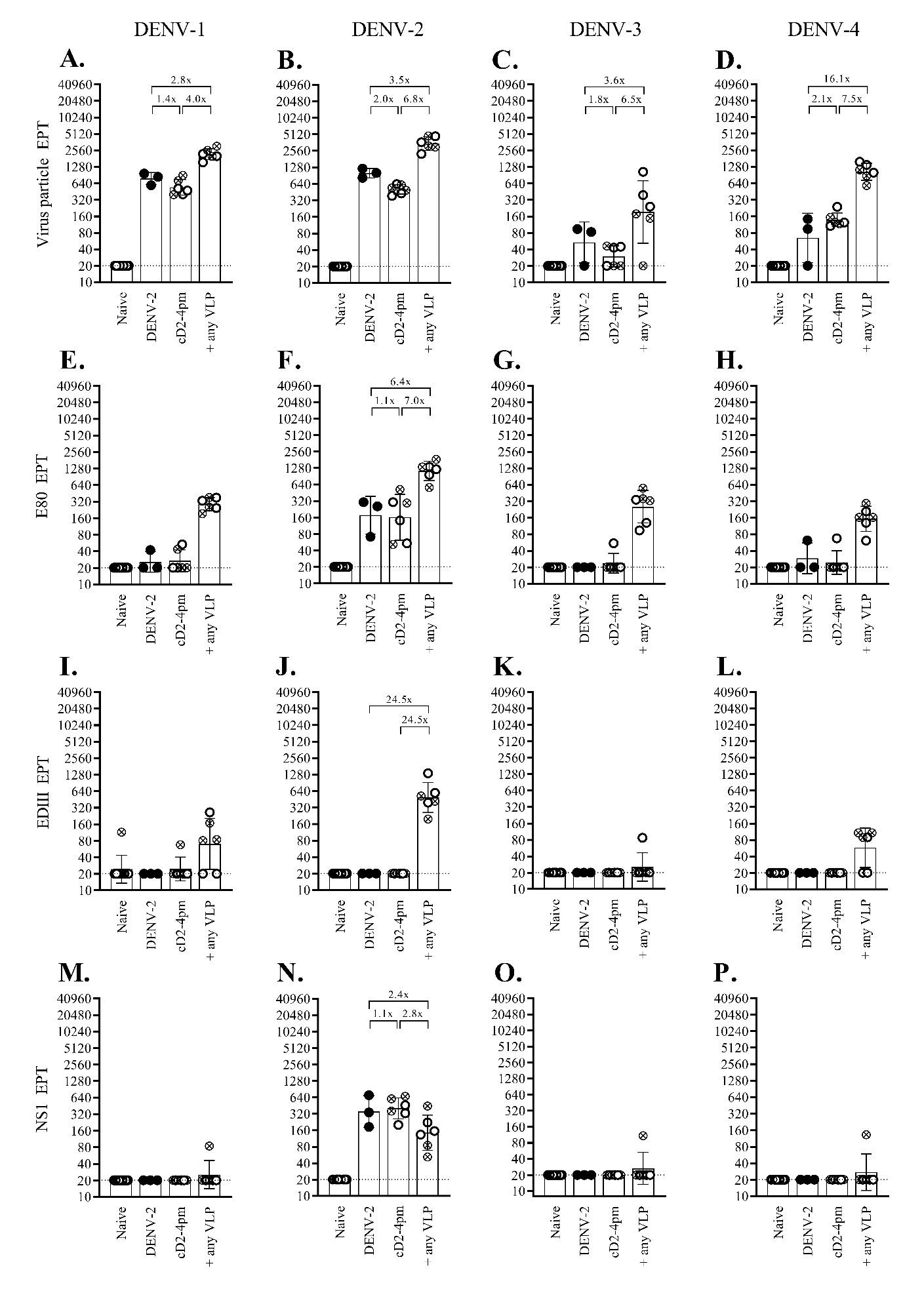


**Fig. S5** Changes in cross-reactive binding antibodies recognizing dengue virus particle, E80 and EDIII fragments of the E protein, and the NS1 protein following DENV-2 infection and related monovalent prime-boost immunization. Groups of three and six macaques were infected with DENV-2 clinical strain 03-0420 (DENV-2) and received the monovalent heterologous prime-boost immunization (cD2-4pm) followed by two doses of DENV-1 pVLP or mVLP (+ any VLP), respectively. Blood samples taken one month after infection or immunization were tested by ELISA for dengue virus particles (**A**–**D**), E80 fragment (**E**–**H**), EDIII fragment (**I–L**), and NS1 protein (**M–P**). Viral particle/proteins derived from each of the four dengue serotypes are indicated at the top. Black circles represent non-immunized control macaques infected with strain 03-0420. Crossed circles represent pVLP-boosted macaques. The limit of detection at the first dilution of sample (1:40) was 20, as shown by the dotted line. The bars and error lines represent geometric means and geometric standard deviations, respectively. The ratios of geometric means were determined with the lower value always set as the denominator. EPT, endpoint titer.


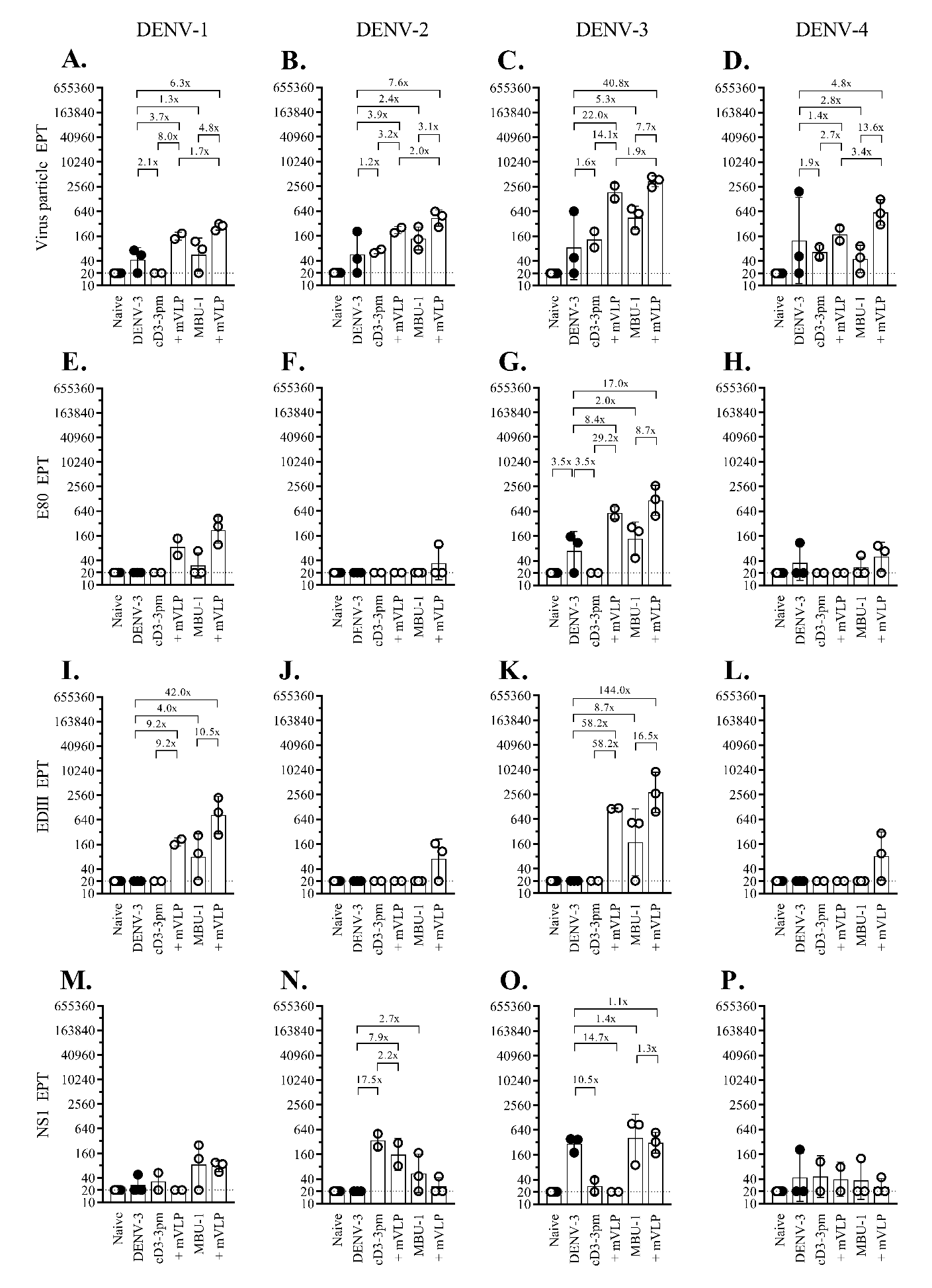


**Fig. S6** Changes in cross-reactive binding antibodies recognizing dengue virus particle, E80 and EDIII fragments of the E protein, and NS1 protein following DENV-3 infection and related monovalent prime-boost immunization. A group of three macaques were infected with DENV-3 clinical strain 06-129 (DENV-3). Additional groups of two and three macaques received strain cD3-3pm (cD3-3pm) and strain MBU-1 (MBU-1), respectively, followed by two doses of DENV-3 mVLP (+ mVLP). Blood samples taken one month after infection or immunization were tested by ELISA for dengue virus particles (**A**–**D**), E80 fragment (**E**–**H**), EDIII fragment (**I–L**), and NS1 protein (**M–P**). Viral particle/proteins derived from each of the four dengue serotypes are indicated at the top. Black circles represent non-immunized control macaques infected with strain 06-129. The limit of detection at the first dilution of sample (1:40) was 20, as indicated by the dotted line. The bars and error lines represent geometric means and geometric standard deviations, respectively. The ratios of geometric means were determined with the lower value always set as the denominator. EPT, endpoint titer.


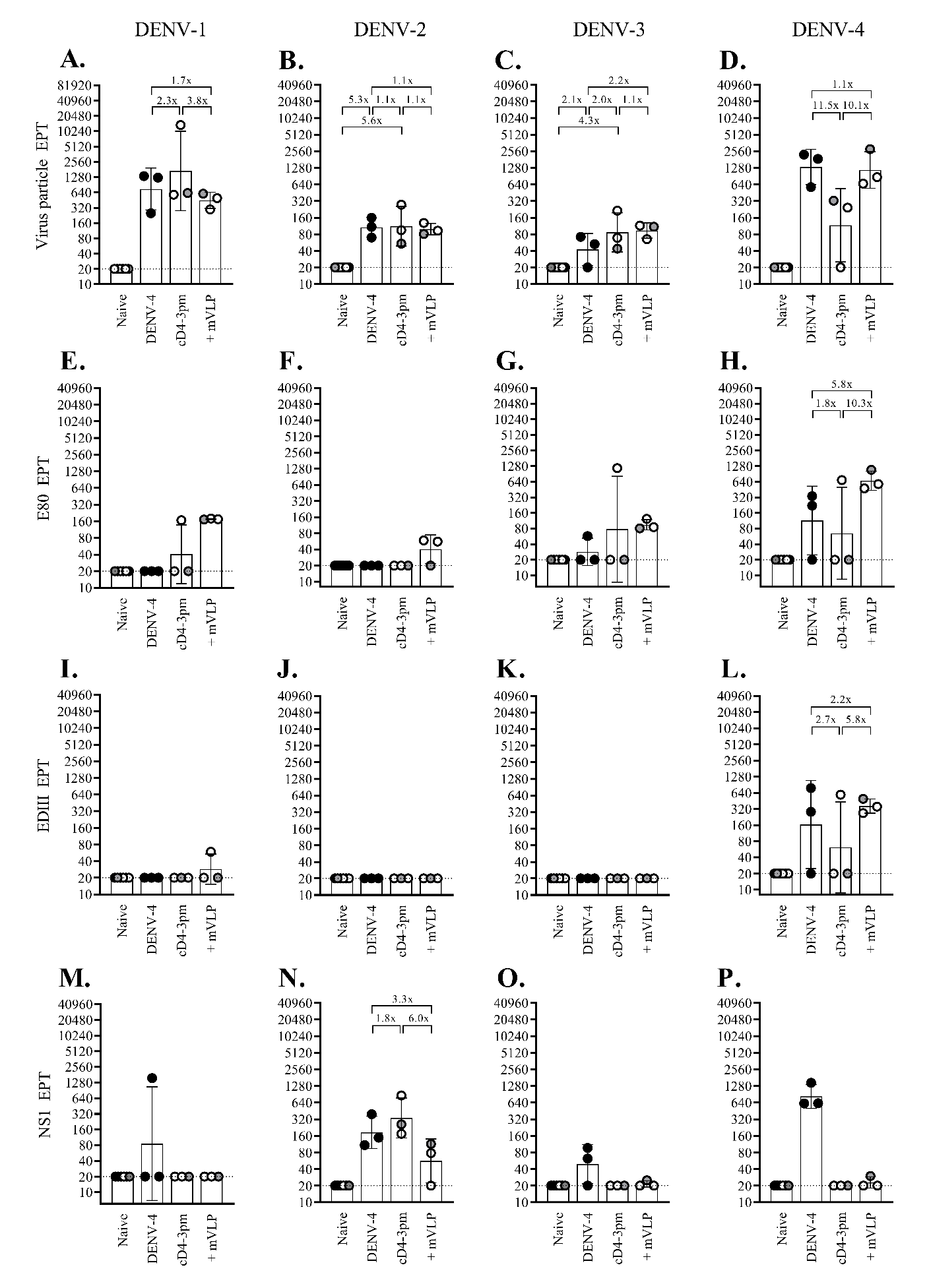


**Fig. S7** Changes in cross-reactive binding antibodies recognizing dengue virus particle, E80 and EDIII fragments of the E protein, and NS1 protein following DENV-4 infection and related monovalent prime-boost immunization. Two groups of three macaques each were infected with DENV-4 clinical strain 2-0201-5 (DENV-4) or received monovalent heterologous prime-boost immunization (cD4-3pm) followed by two doses of DENV-4 mVLP (+ mVLP). Blood samples taken one month (DENV-4) or 45 days (cD4-3pm) after infection or immunization were tested by ELISA for dengue virus particles (**A**–**D**), E80 fragment (**E**–**H**), EDIII fragment (**I–L**), and NS1 protein (**M–P**). Viral particle/proteins derived from each of the four dengue serotypes are indicated at the top. Black circles represent non-immunized control macaques infected with strain 2-0201-5. Empty circles with gray shading represent data from an immunized macaque that was subsequently found to be infected with the challenge DENV-4 virus (macaque `b´). The limit of detection at the first dilution of sample (1:40) was 20, as indicated by the dotted line. The bars and error lines represent geometric means and geometric standard deviations, respectively. The ratios of geometric means were determined with the lower value always set as the denominator. EPT, endpoint titer.


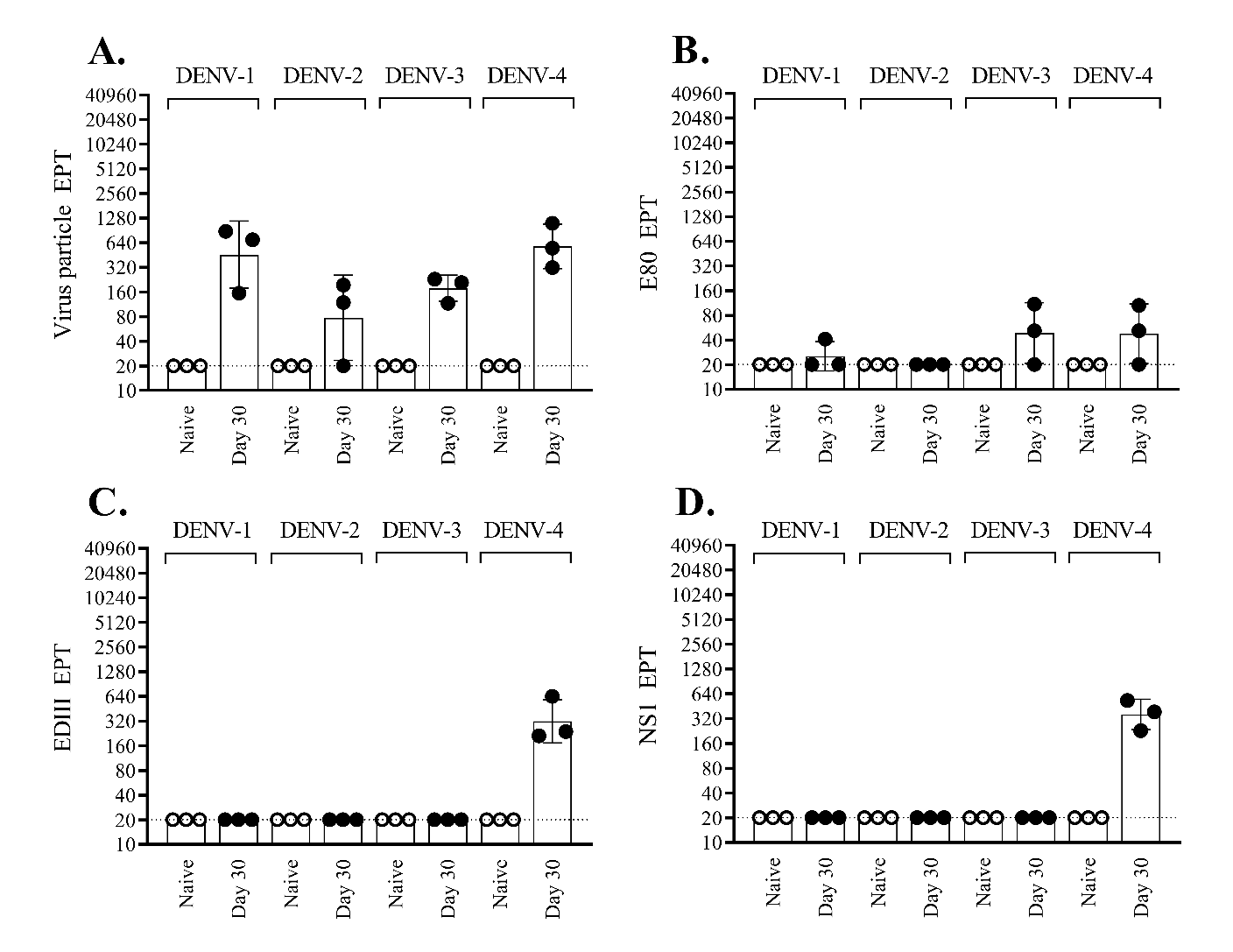


**Fig. S8** Changes in cross-reactive binding antibodies recognizing dengue virus particle, E80 and EDIII fragments of the E protein, and NS1 protein following DENV-4 infection. A group of three macaques was infected with dengue clinical strain 2-0201-5 (DENV-4) at the Indonesian facility. Blood samples taken at baseline (Naïve) and one month (Day 30) after infection were tested by ELISA for dengue virus particles (**A**), E80 fragment (**B**), EDIII fragment (**C**), and NS1 protein (**D**). Viral particle/proteins derived from each of the four dengue serotypes are indicated at the top of each panel. Empty circles represent baseline samples. Black circles represent macaques one month after virus infection. The limit of detection at the first dilution of sample (1:40) was 20, as indicated by the dotted line. The bars and error lines represent geometric means and geometric standard deviations, respectively. EPT, endpoint titer.


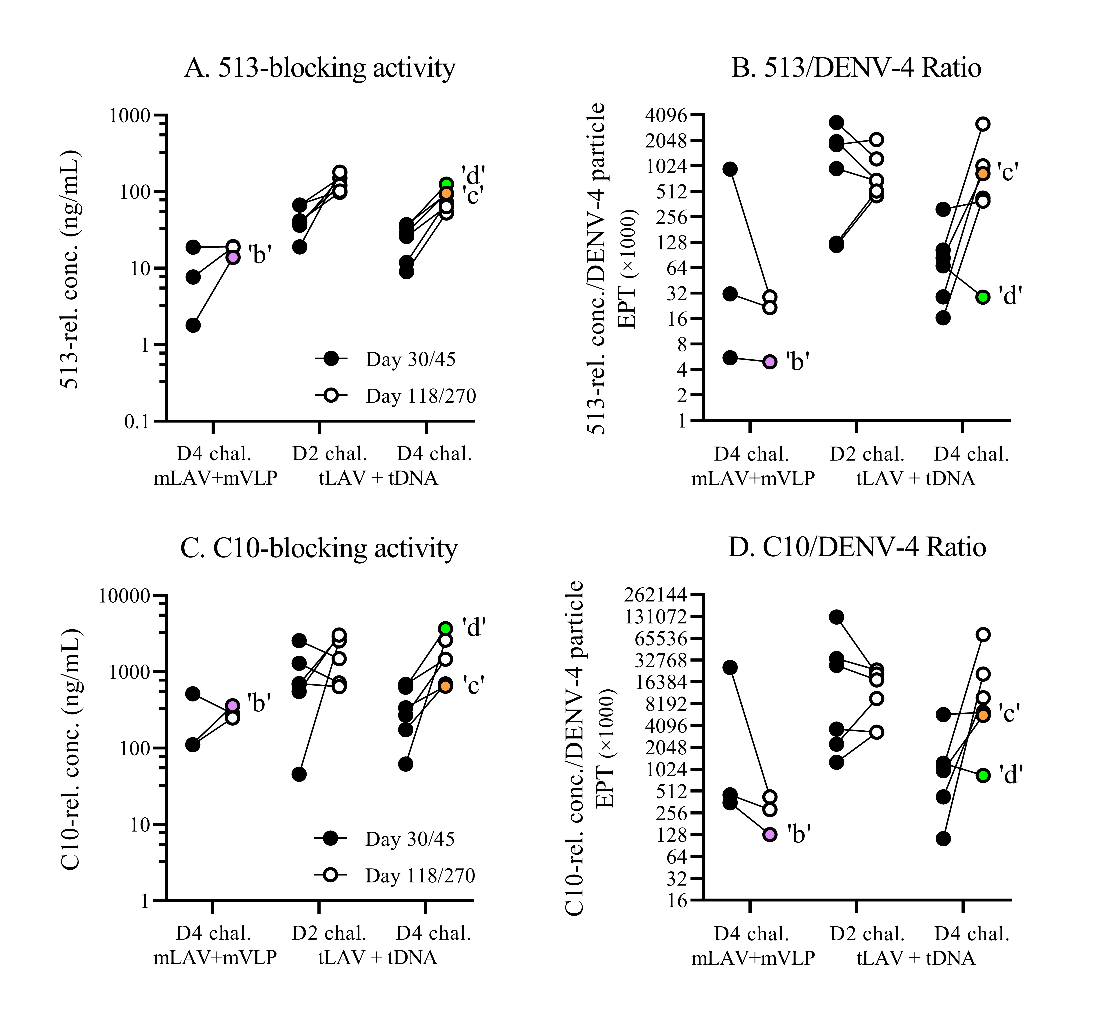


**Fig. S9** Changes in blockade-of-binding activity, virus particle-binding antibody, and blocking/binding ratio during prime-boost immunizations. The relationships of the 513- or EDE-1 C10-blocking activities and the ratios between the blocking activity and DENV-4 particle-binding antibody are shown at the day of peak PRNT titer following the priming LAV injection (Day 30 or Day 45; black circles) and one month after the second boosting injection (Day 118 or Day 270, empty circles) for the DENV-4-related monovalent prime-boost immunization group (D4 chal. mLAV+mVLP) and the two tetravalent prime-boost immunization groups (D2 chal. +tLAV and D4 chal. +tDNA). Circles with colored shading represent macaques that were subsequently found to be infected with the DENV-4 challenge virus: macaque `b´ (purple circle in **A–D**, D4 chal. mLAV+mVLP), macaque `c´ (orange circle in **A–D**, D4 chal. +tDNA), macaque `d´ (green circle in **A–D**, D4 chal. +tDNA).
